# A supine exercise program linking trunk stability with lower extremity coordination is associated with improved body balance and agility: A study using randomized crossover and pre-post trial designs

**DOI:** 10.1371/journal.pone.0345749

**Published:** 2026-04-29

**Authors:** Aya Atomi, Mizuki Sato, Masaki Oyauchi, Wataru Takano, Miho Shimizu, Toshiyuki Watanabe, Tomoaki Atomi, Yoriko Atomi

**Affiliations:** 1 Material Health Science, Graduate School of Engineering, Tokyo University of Agriculture and Technology, Koganei, Tokyo, Japan; 2 Center for Mathematical Modeling and Data Science, Osaka University, Toyonaka, Osaka, Japan; 3 Division of Applied Chemistry, Graduate School of Engineering, Tokyo University of Agriculture and Technology, Koganei, Tokyo, Japan; 4 Physical Therapy Major, Department of Rehabilitation, Faculty of Health Sciences, Kyorin University, Mitaka, Tokyo, Japan; Umm Al-Qura University, SAUDI ARABIA

## Abstract

This study aimed to develop a supine exercise program that promotes coordination between trunk stability and lower extremity control, and to evaluate its short-term effects on balance and physical function in healthy young adults. In human postural control, vertical alignment of the head, thorax, and pelvis—segments with relatively high mass—is essential for biomechanical stability. This alignment is regulated through coordinated activity of the lower limbs in bipedal standing and movement. Thus, exercise interventions that integrate trunk and lower extremity function may improve movement efficiency and postural control. Using an experimental framework modeled on clinical study design, we conducted two separate studies to assess the short-term effects of a supine exercise program designed to link trunk stability with coordinated lower extremity control. The program consisted of a series of movements performed in a supine position to facilitate integrated neuromuscular control between the trunk and lower limbs. Experiment 1 was a randomized crossover trial involving 17 healthy males. Participants completed both intervention and control phases, with physical function assessed via six tests (grip strength, sitting trunk flexion, sit-ups, standing long jump, side-step, and 50-meter run) and static balance evaluated using stabilometry. Experiment 2 used a pre-post design with 22 healthy males and females, assessing dynamic balance through kinematic analysis of the side-step task. In both experiments, participants performed a 10-minute supine coordination-focused exercise program once daily for two weeks. Results showed significant improvements in static balance, side-step kinematics, and sitting trunk flexion. No significant changes were observed in muscle strength or power-related performance tests. These findings suggest that this short-duration exercise program in a biomechanically safe supine position may enhance postural control and flexibility in healthy individuals. The approach may also be useful for fall prevention and early-stage rehabilitation.

Trial Registration

This study was registered with the UMIN Clinical Trials Registry (UMIN000057589 and UMIN000057597).

## Introduction

The multisegmental structure of the body, position of the higher body center of gravity (COG), and influence of the narrow base of support (BOS) composed of both feet are the factors involved in the mechanical stability of human standing and walking [1–3]. Particularly, as the head-to-trunk segment accounts for 50%–60% of the whole-body mass ratio, to maintain standing posture and stability during locomotion, the head-to-trunk segment, which has a high mass ratio, should be properly supported by the two lower extremities [4,5]. The head-to-trunk segment consists of a structure called the axial skeleton, which is composed of the spinal column from the skull to the sacrum and ribs and soft tissues of varying mechanical stability, including the skin and muscle, which are structurally segmental. In maintaining the standing posture, adaptively adjusting according to the situation is performed to stabilize the posture [3,6]. Besides the size of the mass, the trunk comprises several rigid segmental structures, with the thoracic region consisting of the thoracic vertebrae and ribs and the pelvic region consisting of the sacrum and hip bones. It is well known that the COG of the whole body in the upright standing position is located in the pelvis, whereas the thorax, the composite COG of the head, arms, and trunk, approximates the location of the COG of the upper body [7]. In addition, the thorax is a key segment of the body that controls the structural redundancy of the trunk in the standing position [6]. Findings of simulation models also have reported that in human-specific bipedal postural and motor control, instability due to the high COG can be dynamically stabilized by setting a virtual control target at a higher position than the COG [8]. Therefore, in human standing posture control involving instability due to bipedalism, biomechanical stability is obtained by vertically arranging the head, thorax, and pelvis, which are body segments with high mass ratios, and adaptively controlling them according to postural maintenance and movement [9–11]. Thus, with appropriate antigravitational postures and movements, humans stably control these multisegmental structures of the trunk segment, such as the thorax and pelvis, which has a high mass ratio in the body, through the coordinated control of both lower extremities [1,12–16].

The segmental control capacity of the head-to-trunk segment significantly impacts efficiency and stability in activities of daily living [17]. Disruption of segmental control capacity contributes to trunk dysfunction, such as low back pain [18,19], as well as degenerative diseases of the lower extremities resulting from increased biomechanical stress [20,21]. In gait, the hip extensors provide propulsion for movement, and the early stance phase of loading in gait causes the knee, hip, and trunk extensors to collaborate, as well as the ankle joint [22]. The relationship between the segmental control of the head to the trunk and the coordinated function of the lower extremities is represented as the locomotor–passenger relationship, which influences each other [20]. Therefore, for humans to perform effectively in a bipedal upright posture, the segmental properties of the thorax and pelvis—which have high mass ratios—as well as the mechanisms underlying head stability and the coordination of the lower extremities that support them, must be controlled in an integrated manner. This, in turn, facilitates the adaptive regulation of both static and dynamic balance within the limited base of support (BOS) provided by the sole during activities such as standing and walking. Therefore, effectively implementing an exercise program that integrates trunk stability with lower extremity coordination is crucial for maintaining and enhancing activities of daily living and quality of life (QOL).

The effectiveness of exercise programs for improving trunk function has been widely reported. In particular, increasing muscle activity in the transversus abdominis, multifidus, pelvic floor muscles, and diaphragm, known as core muscles, is believed to improve trunk stability [23]. Specifically, the significance of core muscle training is widely recognized in fitness and athlete training [24,25], rehabilitation for cerebrovascular disorders [26,27], and training older adults to improve their standing balance [28,29] and walking stability [30]. Draw-ins, planks, and crunches, which stimulate the contraction of the core muscles of the trunk, are exercise programs that promote trunk stability. Performing a supine breathing exercise followed by a standing task that uses abdominal muscle groups can promote more abdominal contraction [31]. These training exercises have also been incorporated into medical practice, including draw-in and breathing exercises for patients with low back pain. Various approaches to lower extremity exercises have been reported, from athletes to recreational activities for older adults. Resistance training for individual muscle groups in the lower extremities has also been widely used. However, there are also suggestions that the effects of these exercises are limited in terms of standing balance and fall prevention [32,33].

At the same time, exercise programs that improve the coordination of each joint in the lower extremities of the hip, knee, and ankle joints are thought to be more effective in enhancing bodily balance and fall prevention [34]. Several studies have indicated the significance of exercise programs that link them together for improving trunk and lower extremity function [35]. Hodges et al. reported that in healthy individuals, transversus abdominis contraction precedes upper and lower extremity movement, compared with individuals with chronic low back pain [21,22]. Furthermore, Perry et al. stated that in walking, which is an antigravitational activity, the ground reaction force is input only from the sole; therefore, to carry the large mass of the head and trunk forward, activating the muscle groups mainly the tibialis anterior, quadriceps, and gluteus maximus muscles at the appropriate timing during the Initial Contact to the Loading Response phase in the gait cycle is essential [20]. Consequently, enhancing the activity of the trunk muscle group preceding the lower extremity muscle group in response to the ground reaction force is considered effective in stabilizing posture and movement against gravity. Additionally, when rapid displacement of the COG of the whole body in an appropriate direction is required—such as in situations demanding agility—proper movement is achieved through the coordinated control of the segmental body structures with head to trunk and the individual joints of the lower extremities [36–39].

Exercise programs designed to improve static and dynamic balance may place the participant in an unstable condition, including one-legged standing. From a kinematic point of view, posture and movement become unstable when the BOS is narrow, and the COG is high and more likely to become stable when the BOS is wide and the COG is low. Asymmetry in the standing posture affects the body’s balance stability [40]. Furthermore, humans have a complex motor control system that coordinates the activity of a large number of muscles to execute movements or maintain posture [41]. In the relationship between posture and muscle activity, the standing posture requires the activity of antigravity muscles to maintain that posture, and the pattern of that activity affects postural stability [42,43]. Therefore, the standing posture requires complicated balance control and coordinated antigravity muscle activity to control biomechanical instability owing to the high COG in the narrow BOS. Interestingly, several studies have shown that patients with low back pain tend to have higher erector spinae muscle activity in the lumbar back, which are antigravity muscles in the trunk, than healthy controls, suggesting that in poor posture, antigravity muscles are likely to be overworked in daily life [44–46]. Conversely, in the supine position, the BOS is wider, COG height is lower, and posture is biomechanically stable, which may suppress the activity of antigravity muscles in the trunk and lower extremities from kinematics [14].

Based on these findings, we hypothesized that implementing an exercise program that integrates segmented muscle activity of the trunk with coordinated lower extremity function in a stable posture, such as supine, would improve the stability of upright posture and movement safely and in a short duration. However, no studies have shown the effects of such stable, short-duration exercise programs performed in the supine position to improve the linkage between trunk stability and lower extremity coordination for postural stability and movement. To empirically examine this hypothesis, we developed a novel supine exercise program that links trunk and lower extremity function through integrated segmental control. To rigorously assess its effects on physical function and postural control in healthy individuals, we conducted two separate experiments using an experimental framework modeled on clinical study design: a randomized crossover trial and a pre–post trial. To assess program effectiveness, we included both general physical fitness tests and specific kinematic assessments: stabilometry to evaluate static standing posture and tri-axial accelerometry to measure head and trunk stability during side-step movement.

## Methods

### Trial design

**Fig 1:**
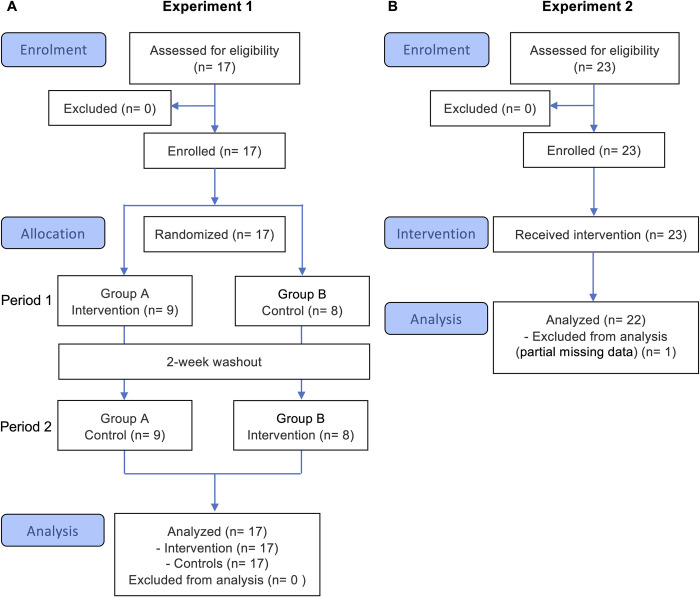
Flow diagram of the study procedures for Experiments 1 and 2.

This study comprises two separate experiments- Experiment 1 (randomized crossover design) and Experiment 2 (pre-post design)- that evaluated the same trunk and lower extremities coordination exercise program in healthy young adults (S1 File). In Experiment 1, the effects of the exercise program were evaluated in 17 healthy males by comparing the intervention and control groups using multiple physical fitness assessments (PFA) and static balance assessments (Fig 1A). In Experiment 2, the effects of the exercise program were evaluated in 22 healthy males and females using kinematic analyses of dynamic balance performance (Fig 1B).

### Exercise program protocols

**Fig 2:**
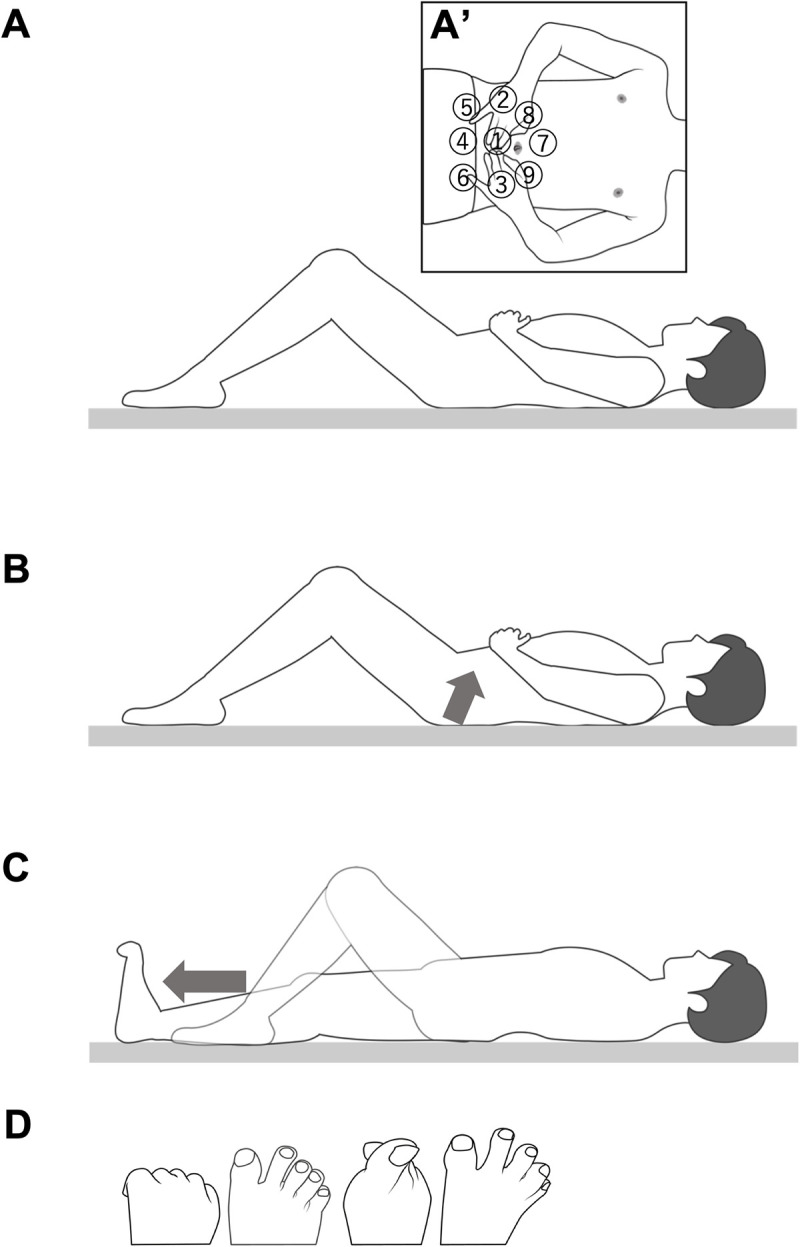
Exercise program protocols. The exercise program comprised the following three types of exercises. (A) This is an exercise of the abdominal muscles. Participants flexed their knee joints, placed their hands on their abdomens, and lightly compressed using their fingertips. They contracted the abdominal muscles in that area against the pressure for 5 s and subsequently relaxed. The inlet shows the abdominal region in the frontal plane (A’). The abdomen is divided into nine areas surrounding the lower end of the ribs, pubis, and both iliac crests. Participants performed the exercise in each area three times (Exercise 1). (B) This is an exercise that improves trunk and lower extremity linkages. Participants flexed their knee joints, with their hands placed on their abdomen. They tilted their pelvis backward while keeping their abdomen contracted and subsequently contracted their hip extensors as in a bridge exercise. The arrow indicates the direction of pelvic movement. They contracted for 5 s with their hips slightly off the floor and subsequently relaxed, repeating ten times (Exercise 2). (C) This is an exercise that improves lower extremity muscle coordination. Participants started in a supine position with both lower extremities extended, then they flexed one knee to 90°, and placed the sole on the floor. Next, they maintained the flexed toe and dorsiflexed ankle joint positions. Subsequently, their hip and knee joints were extended while slightly scraping their heels on the floor while maintaining the angle of the ankle joint as much as possible. The arrow indicates the direction of leg movement. Finally, their lower extremities, with their knees fully extended, were exerted for 5 s by pushing their heels out caudally and subsequently relaxed. Both lower extremities were exercised three times alternately (Exercise 3). (D) From left to right, with both lower extremities in extension, rock-paper-scissors training was performed with the toes: rock (clenching the toes), scissors (raising the thumb), scissors (behind the thumb), and paper (keeping toes apart). This exercise was repeated five times. The figure shows the right toes (Exercise 3).

The exercise program included exercises for improving trunk muscle abilities (Exercise 1, Fig 2A), trunk and lower extremity linkage (Exercise 2, Fig 2B), and lower extremity coordination (Exercise 3, Fig 2C, D), all of which were performed in the supine position.

In each experiment, the same physical therapist instructed the exercise program individually for each participant, both orally and practically, on the first day and 1 week after starting the period in the intervention group. Participants performed one set of the exercise program each day at home while watching a picture and video (the video was provided in Experiment 1) of the exercise program, and they were provided with a checklist to examine whether they performed the exercise, which they submitted 1 week after starting and at the end of the program. The exercise program lasted approximately 10 min. Participants were asked not to change their daily activities and sporting habits during the experimental period.

The exercises shown in Fig 2 were intentionally designed to minimize physical risk, and no harms were associated with their implementation. Harms were defined as falls during measurement sessions and musculoskeletal pain or physical discomfort that interfered with daily activities. Participants recorded their health status daily on paper throughout the study period, and the researchers retained the records. No harms were reported during the study. Participants were allowed to discontinue the study at any time due to physical discomfort, adverse events, or any other reason at their own request.

### Trial registration

This study comprises two separate experiments: Experiment 1 (randomized crossover design) and Experiment 2 (pre-post design). Both experiments evaluated the same trunk and lower extremities coordination exercise program, which was low in intensity and performed in a supine position, in healthy young adults. At the time of study initiation, the authors considered the study to fall outside the scope of a clinical trial, as the intervention was low-risk, short in duration, and conducted in asymptomatic participants, with outcomes limited to physical function measures. Accordingly, both experiments were initially regarded as physiological and biomechanical investigations (Japanese physical fitness test, stabilometer, and accelerometers). Following study completion, and in accordance with current research transparency and reporting standards, the protocol encompassing both experiments was retrospectively registered at the UMIN Clinical Trials Registry [Experiment 1 is UMIN000057589, and Experiment 2 is UMIN000057597]. The authors confirm that all ongoing and related trials for this intervention are registered (S1 File).

### Experiment 1

#### Trial design.

In experiment 1, randomized conditions and crossover designs were conducted to evaluate the effects of the exercise program on several PFAs and static body balance assessments (Fig 1A).

#### Participants.

Seventeen healthy male participants (age, 21.41 ± 0.94 years; height, 170.96 ± 4.37 cm; weight, 60.39 ± 7.17 kg) were included in experiment 1. To verify the effects of coordinated trunk and lower extremity exercises on physical function—including trunk flexibility and agility—we referred to prior outcome data presented either as medians with interquartile ranges [37] or as means with standard deviations [47]. For data in median/IQR format [37], the mean and standard deviation were estimated using the method by [48], implemented in Python. Based on effect sizes estimated from these prior studies, the required sample size was calculated using G*Power 3.1. We assumed a two-tailed paired t-test with α = 0.05, power = 0.9, and a conservative effect size of Cohen’s d = 0.9, chosen to ensure adequate power while maintaining feasibility in this exploratory study. Informed by these parameters, we determined that a minimum of 14 participants would be sufficient. Ultimately, 17 participants were recruited. The following was the exclusion criterion: those with musculoskeletal injuries that prevented them from achieving the exercise program protocol. This experiment was conducted following approval by the Ethics Review Committee of Tokyo University of Agriculture and Technology (TUAT) (approval number: 210901−0335). Participants were recruited through a bulletin board advertisement posted at TUAT between October 12 and November 2, 2021. The experimental intervention was conducted between November 3 and December 16, 2021. All participants voluntarily agreed to participate, received written and oral explanations for the study, and provided written informed consent before participation. The data were collected at TUAT Koganei campus.

#### Interventions.

A randomized crossover trial was conducted to evaluate the effects of the exercise program on physical function and the control of static balance. The experimental design consisted of two intervention periods (Period 1 and Period 2), each lasting two weeks, separated by a two-week washout period (Fig. 1A). Participants were randomly assigned to two groups: Group A (n = 9) and Group B (n = 8). Outcome measures were assessed at the beginning and end of each period. Participant enrollment and group assignment were performed by personnel who were blinded to the random allocation sequence. All participants were managed using anonymized ID codes, and allocation details were accessible only to the individual responsible for randomization. Although the study was open-label, outcome assessors were blinded to group assignments to minimize bias. The intervention was conducted in accordance with the exercise protocol detailed in Fig 2.

#### Outcomes.

The following were the PFAs at the beginning and end of periods 1 and 2: grip strength, sitting trunk flexion, sit-ups, standing long jump, side-step, and 50-m run. These PFA items are common PFAs in Japan. They are collectively called the New Physical Fitness Test by the Ministry of Education, Culture, Sports, Science and Technology and are conducted nationwide for elementary through junior high school students [49]. Also, the static balance of upright standing was assessed using stabilometry.

### Measurements and procedures

#### Side-step.

**Fig 3:**
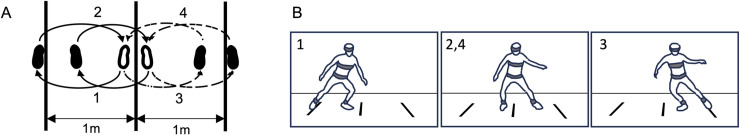
Procedure for the side-step test. (A) In the side-step test, three parallel lines are marked on the floor at 1-meter intervals. Participants start by straddling the center line (indicated by the white footprints). They then perform the side-step (black footprints) in the sequence shown by the numbered arrows (1–4), completing one full cycle. The participant repeats this cycle—stepping to the side and returning to the center—as many times as possible within 20 seconds. (B) Snapshots of the side-step motion. The numbers correspond to the arrows shown in panel A.

Side-step was measured to evaluate agility, a component of dynamic balance. In side-step, three parallel lines were drawn on the floor at 1-m intervals, and the front-facing position and straddling the center line were the starting positions (Fig 3). Participants started at the sound cue, sidestepped laterally until their feet crossed or stepped on the right (or left) line, and subsequently repeated the cycle of stepping to the center line and returning as many times as possible in 20 s. The step count was measured as the number of times the participant stepped or crossed the left, right, and center lines. The measurements were taken after sufficient practice (Fig 3).

#### Grip strength.

To evaluate whole-body muscle strength, grip strength was measured [50,51] using a Smedley grip strength meter (103-KS, Hata Sporting Goods, Higashiosaka, Japan). Participants were instructed to grip to the maximum in an upright position, with both feet hip-width apart, arms lowered, and not touching the grip meter to their body or clothing. Measurements were taken two times, alternating left and right, and the average value was calculated using the higher value of each.

#### Sitting trunk flexion.

To evaluate flexibility, sitting trunk flexion was measured. As the starting position, participants sat on the floor with their backs on the wall and their knees extended, and a digital sitting trunk flexion meter on casters (T.K.K5412, Takei Scientific Instruments, Kamo, Japan) was placed wherein both upper extremities were reached forward without bending the elbows, and both hands were placed on it as the reference position. Participants were asked to slowly bend their trunks forward without bending their knees and slide the meter as far forward as possible without removing their hands from the digital sitting trunk flexion meter. Measurements were taken twice as the length moved when flexing forward to the maximum, and the longer recorded measurement was used.

#### Sit-ups.

To evaluate the endurance of the trunk muscles, sit-ups were measured. Participants were instructed to lie supine on a mat with their arms crossed in front of their thorax, hands slightly grasped, and knees flexed at 90°. An assistant held the participant’s knees and fixed them in position. On cue to begin, the participant was asked to lift his/her upper body from a supine position with both shoulder girdles on the mat until both elbows and thighs touched each other, repeating as many times as possible in 30 s. The measurement was taken once from supine to back again.

#### Standing long jump.

To evaluate the explosive power of the lower extremities, standing long jump was measured. Participants stood on the line of the floor with their feet hip-width apart and jumped forward as far as possible at their timing. Measurements were taken twice. The longer distance from the line to the point of landing was recorded.

#### Fifty-m run.

To evaluate whole-body explosive power, 50-m run was measured. A time measuring device (FASTRun, Y.Y. Factory, Kobe, Japan) was attached to the trunk. The participants stood on a line drawn on the ground, and the time from the start signal, until their trunk reached the measurement device at the goal line was measured. Measurements were taken over two runs, and the shorter time was recorded.

#### Static balance of upright standing.

To evaluate the static balance of upright standing, a stabilometer (T.K.K. 5810, TAKEI Scientific Instruments, Kamo, Japan) was used. COG sway was measured using standard methods [52]. Measurements included a vertical force plate for measuring instantaneous center of pressure (COP) fluctuations; the COP signal was sampled at 20 Hz and stored on a personal computer.

As the starting position, each participant stood in a relaxed upright posture on the stabilometer, with a marker at eye level on the wall 2 m. The following were the two standing conditions: natural standing, wherein each participant stood comfortably, with slightly spread legs, and joined parallel standing [53,54]. The order of the two standing conditions was computer-randomized; both were performed with open eyes, and measurements were taken for 30 s per condition. The stabilometer was used for measuring the COP, and the total locus length (TLL) and sway area (SA) were calculated using the software for stabilometer (TAKEI Scientific Instruments, Kamo, Japan).

#### Data analysis.

To examine the carry-over effect, a paired t-test was first performed on the pre-values in periods 1 and 2 for each PFA item and static balance item respectively (α = 0.05). Subsequently, if the carry-over effect of each item was rejected, they were compared with the pre- and post-exercise program changes, with 17 participants in the control groups and the 17 participants in the intervention groups.

For each item, we calculated either the mean and standard deviation or the median and interquartile range, depending on the distribution. We also calculated the percentage change (Δ%) from the pre- to the post-period. The Shapiro–Wilk test was used to assess the normality of each item. Paired t-tests were applied to normally distributed items, while the Wilcoxon signed-rank test was used for non-normally distributed items.

For the stabilometry-based static balance measures in two items, sway area (SA) and total locus length (TLL), we performed comparisons under two stance conditions: natural standing and joined parallel standing. For the pre–post comparisons, a total of four tests (two stance conditions and two groups) were conducted, and Bonferroni correction was applied separately within each balance item, resulting in a corrected significance threshold of α = 0.0125. Additionally, for the between-group comparisons (Control vs. Intervention) of the pre-post differences within each stance condition, two tests were performed for each item, and Bonferroni correction was applied within each item, setting the corrected threshold at α = 0.025. This procedure ensured that multiple testing was controlled independently for SA and TLL. All statistical analyses were performed using JASP v. 0.19.1 (Univ. of Amsterdam, Netherlands).

### Experiment 2

#### Trial design.

In experiment 2, a pre–post interventional study was conducted to evaluate the effects of the exercise program on kinematics in dynamic balance (Fig 1B).

#### Participants.

Twenty-three healthy male and female participated in experiment 2, but one male was excluded due to partial missing data (age 22.23 ± 1.41 years, height 172.17 ± 8.30 cm, weight 65.33 ± 11.68 kg, 19 males and 3 females). To examine the effects of the exercise program on trunk kinematics during agility tasks, we used prior outcome data reported either as medians with interquartile ranges [37] or as means with standard deviations [55]. As in Experiment 1, data reported as medians and IQRs [37] were converted to estimated means and standard deviations using the method of Hozo [48], implemented in Python. Effect sizes estimated from these sources informed our sample size calculation using G*Power 3.1, assuming a two-tailed paired t-test with α = 0.05, power = 0.9, and a conservative Cohen’s d = 0.9. These calculations indicated a required sample size ranging from 14 to 23, depending on the specific outcome. Based on this estimation, we recruited 22 participants for Experiment 2.This experiment was conducted following approval by the Ethics Review Committee of TUAT (approval number: 191106−3130). Participants were recruited through a bulletin board advertisement posted at TUAT between October 29 and November 15, 2019. The experimental intervention was conducted from November 16 to December 18, 2019. The unit of assignment was the individual. All participants voluntarily agreed to participate, received written and oral instructions for the study, and provided written informed consent before participation. The data were collected at TUAT Koganei campus.

#### Interventions.

Participants performed a two-week exercise program. The instructional procedures were the same as in Experiment 1 and were implemented in accordance with the protocol outlined in Fig 2.

#### Outcomes.

Outcome measures were obtained before and after the two-week intervention period. For the kinematic analysis of the side-step, three-axis accelerometers were attached to the head, thorax, pelvis, and lower extremities to measure the acceleration in each body segment during the task.

### Measurements and procedures

#### Side-step.

In side-step, acceleration at each body segment was measured. A small wireless multifunctional sensor (TSND-151, ATR-Promotions, Japan) was affixed and banded to the participant’s head (at the occipital external protuberance), thorax (at the eighth thoracic spinous process), pelvis (at the second sacral thoracic spinous process), and bilateral lateral malleolus, and acceleration in the three axial directions was measured at each location. A software (ALTIMA, ATR-Promotions, Kyoto, Japan) was used for measurement and analysis, with a 50-Hz sampling frequency and a range of ± 8 G, and a resolution of 0.004 G/LSB (16-bit output). The sensor (46 × 46 × 18 mm, approximately 22 g) was connected to a laptop PC in a synchronized system via Bluetooth, and a camera (WEB Cam-c920r, Logitech, Lausanne, Switzerland) was used on ALTIMA to capture videos at 60 FPS from the front.

#### Data analysis.

In side-step, 20 s of the task execution section was analyzed. The timing of the task start was determined from the audio data at the start of the video at task implementation.

The tri-axial resultant acceleration (RA) in the segment was used in the analysis to evaluate the impact produced at the segment using accelerometers [56].

The data were processed as follows: initially, the resultant acceleration (RA) was computed using the acceleration components along the superior-inferior (X), medial-lateral (Y), and anterior-posterior (Z) axes for each time series. Subsequently, a fourth-order Butterworth filter with a cutoff frequency of 20 Hz was applied to perform low-pass filtering. After that, the total resultant acceleration (TRA) was calculated from the summed RA in 20 s of the task section and used as the representative value of each body segment.


$$TRA\;=\;\sum_{i=\text{1}}^{n}\sqrt{{Xi}^{\text{2}}+{Yi}^{\text{2}}+{Zi}^{\text{2}}}$$

The TRA at each body part per side-step (TRA/step) was calculated as follows:


TRA/step = (TRA)/count of side−step

This study evaluated the kinematic changes in each segment during side-step using TRA and TRA/step at the head, thorax, pelvis, and lower extremities pre- and post-exercise program. MATLAB R2024a (Mathworks Inc., USA) was used for all data processing.

#### Statistical methods.

To evaluate the side-step task, pre–post differences were statistically tested for the number of steps as well as for TRA and TRA/step in each body segment. Each variable was tested for normality using the Shapiro–Wilk test (α = 0.05). When normally distributed, paired t-tests (α = 0.05) were applied; otherwise, Wilcoxon signed-rank tests (α = 0.05) were used. Bonferroni correction was applied to adjust for multiple comparisons within the kinematic outcomes, with axial segments (head, thorax, pelvis) corrected using α = 0.017 and bilateral lower extremities (right and left) using α = 0.025. All statistical analyses were performed using JASP v. 0.19.1 (Univ. of Amsterdam, Netherlands).

## Results

### Experiment 1

In experiment 1, the carry-over effects for each PFA and static balance item were not statistically significant; therefore, we proceeded to compare the pre- and post-intervention changes in the control and intervention groups. A total of 17 participants were analyzed in each group.

#### PFA.

In the control group, of the six PFA items, only the sit-up test (p = 0.027) was significantly higher, and no significant differences were observed in the other measures at the post-exercise program (Table 1). The intervention group showed significantly higher for sitting trunk flexion (p < 0.001), sit-ups (p < 0.001), and side-step (p = 0.008) at the post-exercise program. The intervention group showed a significantly higher Δ% of each variable pre- and post-exercise program than the control group for sitting trunk flexion (p = 0.002) and side-step (p = 0.02) (Table 1).

**Table 1:** Values for physical fitness assessments (PFAs) and body balance assessment (static balance of upright standing) at the pre- and post-exercise program (n = 17) in experiment 1.

				Pre versus Post				Control versus Intervention			
				Mean	SD	P value	Effect size	Mean	SD	P value	Effect Size
				Median	IQR		(d, r)	Median	IQR		(d, r)
								(Δ%)			
**Physical fitness assessments (PFAs)**											
**Grip strength**		**Control**	**pre**	39.29	6.62	0.088	0.441	**-2.86**	**-5.71, 0**	**0.201**	**0.383**
**(kg)**			**post**	37.88	6.08						
		**Intervention**	**pre**	39.00	6.62	0.277	0.265	**-1.25**	**6.10**		
			**post**	38.38	6.04						
**Sitting trunk flexion**		**Control**	**pre**	39.53	9.52	0.419	0.222	**4.45**	**13.83**	**0.002** ^**†**^	**0.804**
**(cm)**			**post**	38	34, 43						
		**Intervention**	**pre**	37.82	11.17	< 0.001^***^	1.119	**11.76**	**3.39, 0**		
			**post**	42.35	9.11						
**Sit-ups**		**Control**	**pre**	24.06	5.06	0.027^*^	0.590	**3.70**	**0, 13.33**	**0.339**	**0.279**
**(number)**			**post**	26.12	5.35						
		**Intervention**	**pre**	24.35	5.34	< 0.001^***^	1.225	**10.66**	**8.62**		
			**post**	26.88	5.90						
**Standing long jump**		**Control**	**pre**	214.59	27.17	0.733	0.100	**-0.52**	**5.56**	**0.965**	**0.011**
**(cm)**			**post**	223	200, 233						
		**Intervention**	**pre**	219	206, 230	0.517	0.184	**-0.61**	**4.13**		
			**post**	222	188, 228						
**Side-step**		**Control**	**pre**	59	55, 59	1.000	0.000	**0.00**	**-3.51, 1.89**	**0.02^†^**	**0.634**
**(number of step)**			**post**	56.53	5.59						
		**Intervention**	**pre**	54.47	8.10	0.008^††^	0.757	**5.36**	**3.33, 8.06**		
			**post**	60	55, 61						
**50-m run**		**Control**	**pre**	8.08	0.47	0.121	0.397	**0.80**	**2.01**	**0.100**	**0.423**
**(second)**			**post**	8.14	0.51						
		**Intervention**	**pre**	8.15	0.59	0.658	0.110	**-0.20**	**1.75**		
			**post**	8.13	0.61						
**Static body balance**											
**Sway area of Stabilometer**	**Natural standing**	**Control**	**pre**	116.08	59.5	0.649	0.113	**13.80**	**55.26**	**0.140**	**0.377**
**(SA)**			**post**	109.79	42.9						
**(mm2)**		**Intervention**	**pre**	103.21	38.4	0.193	0.330	**-11.53**	**30.87**		
			**post**	90.62	44.1						
	**Joined parallel standing**	**Control**	**pre**	272.44	129.9	0.605	0.128	**-8.83**	**-29.46, 20.52**	**0.011** ^*^	**0.686**
			**post**	256.52	75.1						
		**Intervention**	**pre**	309.81	132.1	0.003^*^	0.859	**-32.73**	**-39.93, -22.68**		
			**post**	221.93	93.9						
**Total locus length of Stabilometer**	**Natural standing**	**Control**	**pre**	136.04	32.4	0.635	0.117	**7.21**	**29.25**	**0.329**	**0.281**
**(TLL)**			**post**	140.36	32.4						
**(mm)**		**Intervention**	**pre**	136.82	30.8	0.368	0.225	**-6.93**	**-16.58, 1.72**		
			**post**	130.86	35.8						
	**Joined parallel standing**	**Control**	**pre**	209.77	38.5	0.392	0.214	**6.08**	**21.48**	**0.016** ^*^	**0.652**
			**post**	218.38	40.0						
		**Intervention**	**pre**	221.84	41.6	0.004^*^	0.823	**-9.97**	**12.04**		
			**post**	198.32	39.6						

Each measurement is presented as mean and standard deviation or median and interquartile range, according to the results of the Shapiro–Wilk test. For each assessment, the percentage change (Δ%) in the post-exercise program relative to the pre-exercise program is expressed as a percentage. Paired t-test: statistic (t-value), effect size (Cohen’s d, d), * p < 0.05, ** p < 0.01, *** p < 0.001. Wilcoxon singed-rank test: statistic (z-value), effect size (rank–biserial correlation, r), † p < 0.05, †† p < 0.01. For the stabilometry data (sway area [SA] and total locus length [TLL]), Bonferroni correction was applied: for the pre–post comparisons, a corrected significance threshold of α = 0.0125 was used; for comparisons of change scores (ΔPre–Post) between the Control and Intervention groups within each stance condition, the corrected threshold was set at α = 0.025. SD, standard deviation; IQR, interquartile range; diff, differences.

#### Static balance of upright standing.

In the control group, no significant difference in the SA and TLL was noted between the pre- and post-exercise program in both natural standing and joined parallel standing conditions (Table 1). Conversely, in the intervention group, the SA (p = 0.003) and TLL (p = 0.004) were significantly lower in the post-exercise program in the joined parallel standing condition. Regarding the Δ% pre- and post-exercise program, the intervention group had significantly lower SA (p = 0.011) and TLL (p = 0.016) than the control group only in the joined parallel standing condition.

### Experiment 2

#### Number of side-step.

The average and standard deviation of the numbers of side-step were 45.36 ± 7.17 and 48.63 ± 6.56 in the pre- and post-exercise program, respectively (S2 Data). The average of the side-step significantly increased after the exercise program (t (21) = −4.83, p < 0.001, d = 1.03).

#### TRA and TRA/step.

**Fig 4:**
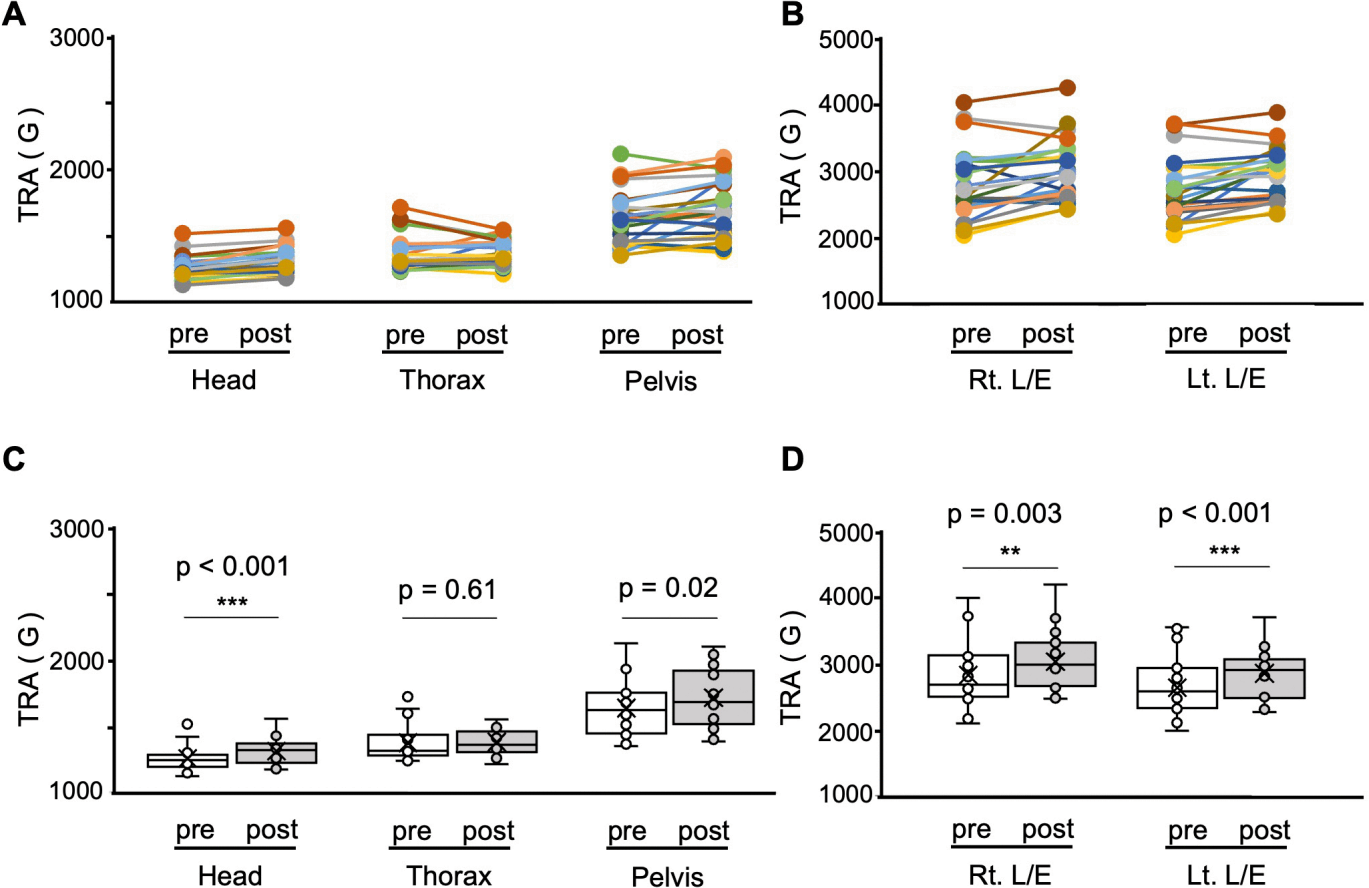
Changes in total resultant acceleration (TRA) during the side-step in Experiment 2. (A) Individual pre–post changes in TRA (G) over a 20-second period at the head, thorax, and pelvis. (B) Individual pre–post changes in TRA (G) at the right and left lower extremities (Rt. L/E and Lt. L/E). (C) Group pre–post differences in TRA (G) at the head, thorax, and pelvis presented as box plots. P-values for each segment are indicated in the figure. (D) Group pre–post differences in TRA (G) at the Rt. L/E and Lt. L/E presented as box plots. P-values for each segment are indicated in the figure. The vertical axis in all panels represents TRA (G). Asterisks (*, **, ***) indicate statistically significant differences between pre- and post-intervention values based on paired t-tests. Bonferroni correction was applied to account for multiple comparisons, with corrected significance thresholds set at α = 0.017 for axial segments (head, thorax, pelvis) and α = 0.025 for lower extremity segments (right and left legs). Accordingly, the asterisk symbols (*p < 0.05, **p < 0.01, ***p < 0.001) represent significance determined in relation to these corrected thresholds.

The TRAs of the head, thorax, pelvis, and lower extremities during the side-step are shown in Fig 4. The TRA of the post-exercise program showed significantly higher than the pre-exercise in the following segments: head, t (21) = −4.74, p < 0.001, d = 1.01; right lower extremity, t (21) = −3.30, p = 0.003, d = 0.70; and left lower extremity, t (21) = −3.93, p < 0.001, d = 0.84. No significant difference were noted in the following: thorax, z (21) = −0.54, p = 0.61, r = 0.13; and pelvis, t (21) = −2.53, p = 0.02, d = 0.54.

**Fig 5:**
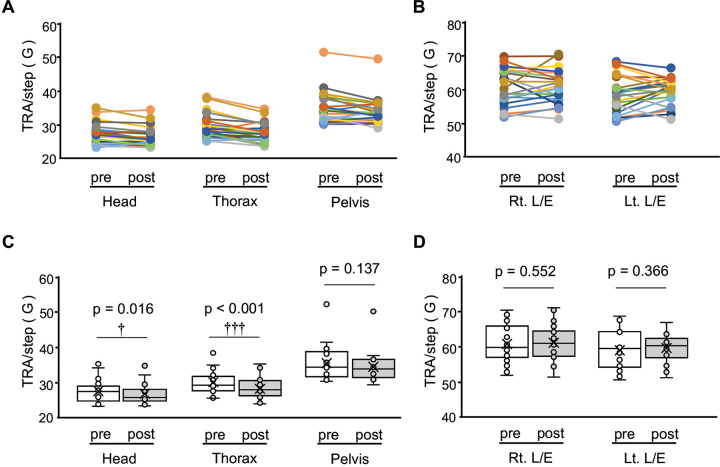
Changes in total resultant acceleration per step (TRA/step) during the side-step in Experiment 2. (A) Individual pre–post changes in TRA/step (G/step) over a 20-second period at the head, thorax, and pelvis. (B) Individual pre–post changes in TRA/step (G/step) at the right and left lower extremities (Rt. L/E and Lt. L/E). (C) Group pre–post differences in TRA/step (G/step) at the head, thorax, and pelvis presented as box plots. P-values for each segment are indicated in the figure. (D) Group pre–post differences in TRA/step (G/step) at the Rt. L/E and Lt. L/E presented as box plots, with p-values for each segment indicated in the figure. The vertical axis in all panels represents TRA/step (G/step). Daggers (†, ††, †††) indicate statistically significant differences between pre- and post-intervention values based on Wilcoxon signed-rank tests. Bonferroni correction was applied to account for multiple comparisons, with corrected significance thresholds set at α = 0.017 for axial segments (head, thorax, pelvis) and α = 0.025 for lower extremity segments (right and left legs). Accordingly, the dagger symbols (†p < 0.05, ††p < 0.01, †††p < 0.001) represent significance determined in relation to these corrected thresholds.

The results of the TRA/step are shown in Fig 5. The TRA/step of the post-exercise program showed significantly lower than the pre-exercise in the following segments: head, z (21) = 2.38, p = 0.016, r = 0.58; thorax, z (21) = 3.36, p < 0.001, r = 0.82. However, no significant differences were noted in the following segments: pelvis, z (21) = 1.51, p = 0.137, r = 0.37; right extremity, t (21) = −0.60, p = 0.552, d = 0.12; and left extremity, t (21) = −0.92, p = 0.366, d = 0.20.

## Discussion

Here, we demonstrated that the supine exercise program led to significant improvements in flexibility, static standing balance, and agility (side-step performance), providing evidence for its effectiveness in enhancing multiple aspects of physical function. In Experiment 1, participants in the intervention group showed greater gains in flexibility (long sitting trunk flexion), agility (side-step test), and static balance performance as reflected by reductions in sway area (SA) and total locus length (TLL), compared with the control group (Table 1). In Experiment 2, analysis of kinematic outcomes during the side-step task further revealed more efficient use of the trunk, particularly improved stability of the head and thorax segments, supporting the interpretation that the exercise program enhanced central stability and movement efficiency during dynamic balance tasks(Figs 4 and 5).

First, the results of improved flexibility will be discussed (Table 1). In this study, the lower extremity coordination program included in the exercise program was designed to model the muscle contraction pattern of the loading phase from heel contact during walking. It involves ankle joint dorsiflexion and knee extension, which causes muscular contraction of the tibialis anterior and quadriceps muscles while stretching the hamstrings and triceps [7]. Additionally, the program facilitates the contraction of the abdominal muscle groups. From a physiological perspective, owing to reciprocal innervation, the inhibitory impulse to the antagonist muscle that accompanies agonist muscle contraction promotes antagonist muscle relaxation [57,58]. This finding suggests that the improvement in sitting trunk flexion can be caused by the stretching of the hamstrings and triceps muscles and the trunk extensor muscle group because of the exercise program.

Second, the results of improved static standing balance ability will be discussed (Table 1). It is known that foot width affects the stability of static standing, and joined parallel standing is unstable due to the narrower support prescriptive surface compared to natural standing [52,53]. In static balance, the base of support (BOS) is a fundamental biomechanical determinant of postural stability [5]. When the BOS is restricted, postural control increasingly relies on strategies such as the ankle and hip strategies and intersegmental coordination to confine the displacement of the center of mass of the whole body within the smaller support area [5,10,59]. Narrowing the BOS, as in joined parallel standing, restricts the range of COM displacement and increases sway area and path length, reflecting greater demands on sensory integration and neuromuscular control [60,61]. Under such constrained conditions, postural control shifts from primarily relying on the ankle strategy toward greater involvement of the hip strategy, reflecting the need for more proximal and segmental control [59]. In the present study, no significant changes were observed under natural standing, but both SA and TLL were significantly reduced after the intervention in the joined parallel standing condition. In the intervention group, these reductions can be interpreted as effects of the exercise program: improved toe function may have effectively increased the base of support, while enhanced trunk–lower extremity coordination facilitated more segmental control of posture. These adaptations may have contributed to more efficient neuromuscular regulation under narrow BOS conditions, possibly facilitating ankle strategy engagement.

Third, the results of dynamic balance will be discussed (Figs 4 and 5). In experiments 1 and 2, the number of side-steps, a measure of agility, was significantly improved in the intervention group compared with the control group. Agility is the ability to move a body part quickly, such as the body as a whole or an extremity, to transfer the bodily position or change the direction of body movement, and is involved in motor control [36]. The results of each TRA in the head, thorax, pelvis, and lower extremities during side-step are depicted in Fig 4. Reductions in TRA/step were observed in the axial segments, particularly in the head and thorax, while no significant change was noted in the thorax and pelvis, despite the overall increase in side-steps. During walking, the RA of the upper body is generally smaller than that of the lower extremities [62], and a similar pattern was observed in the present side-step task, with higher TRA in the lower extremities. The notable finding here is that TRA per step decreased in the head and thorax after the intervention. This suggests that the exercise program improved central stability and movement efficiency by enabling participants to stabilize high-mass axial body segments under dynamic conditions. Previous studies have suggested that achieving bipedal locomotor control in humans is primarily dependent on head stabilization strategies, with interactions between the head and trunk, and between the head and pelvis, causing trunk oscillatory movements to decay before reaching the head [63]. In addition, older adults also prioritize head stability when responding to lateral perturbations, underscoring its fundamental role in maintaining dynamic balance [64]. Furthermore, thoracic control plays a pivotal role in damping high-amplitude trunk oscillations before they reach the head, thereby facilitating head stability during dynamic tasks [11,65]. In particular, the thorax, as a high-mass segment, contributes significantly to maintaining trunk balance in the frontal plane, which in turn stabilizes the head and reduces the corrective burden on the postural system [66]. These findings collectively suggest that the exercise program in this study effectively enabled participants to move like a pendulum with the thorax centered, and stabilizing central body segments such as the head and thorax is a crucial mechanism for efficient movement control during bipedal dynamic tasks. From the perspective of movement efficiency, lower accelerations per step in the head and thorax suggest that participants were able to stabilize axial body segments with fewer corrective actions, thereby reducing the energetic demands required for the movement. Some evidence from studies that have investigated the influences of trunk stability on movement supports this interpretation: impaired trunk dynamic stability in individuals with low back pain compromises efficient postural control [67], and targeted training such as kayak ergometer exercise enhances dynamic trunk stability and efficiency even in paraplegics [68]. In line with these findings, the present reduction in TRA/step likely reflects more economical and coordinated multi-segmental control, achieved mainly through improved stability in the head and thorax. Taken together, the exercise program in this study may have enabled participants to adopt a pendulum-like movement pattern centered on the thorax, potentially allowing efficient lateral transitions of the upper body with its high mass ratio. Furthermore, minimizing excessive head movement in line with the “head stabilization strategy” is thought to have formed the foundation for maintaining dynamic balance [63,69,70]. The design of the exercise program—including the activation of abdominal muscles (Exercise 1), the promotion of coordinated trunk–lower extremity linkage (Exercise 2), and the coordination of muscle contractions during heel strike together with toe muscle activation (Exercise 3)—is thought to have contributed to the observed improvement in agility by optimizing the interaction between axial stability and lower extremities coordination [71].

The exercise program conducted in this study was effective in improving both static and dynamic balance control. To our knowledge, however, no prior research has evaluated an exercise program that is short in duration, performed entirely in the supine position, and specifically designed to be conducted in that posture, as in the present study. In this study, despite the program’s supine nature and short duration, the two-week intervention effectively improved agility, flexibility, and standing balance stability. It is known that muscle hypertrophy requires intensity training to be performed set to voluntary failure [72]. No significant changes were observed in the PFA items that require increased maximal muscle strength, such as grip strength and standing long jump. Therefore, the improved performance was suggested to be not attributable to muscle hypertrophy but rather to a modification within the central nervous system [73–76]. The exercise program performed in this study was a low-intensity supine program conducted over two weeks. Despite its short duration, the intervention yielded measurable benefits in flexibility, repetitive lateral stepping, and static standing balance. Previous research has shown that, after two weeks of moderate isometric training, up to 80% of the observed strength gains can be attributed to neural factors rather than muscle hypertrophy [77]. Short-term interventions in healthy adults have also been reported to enhance motor unit mobilization and early firing, reduce cortical inhibition, and decrease antagonist co-contraction [78–80]. Taken together, these findings suggest that the improvements observed in the present study are likely underpinned by short-term neural adaptations, including changes in motor unit recruitment and reduced antagonist inhibition, rather than structural muscle hypertrophy. Accordingly, the two-week duration, although insufficient to induce morphological changes, appears sufficient to elicit meaningful neuromuscular adaptations that enhance flexibility, coordination, and balance performance. The most distinctive aspect of the exercise programs in this study is that all programs were set up in the supine. In the supine position, it is suggested that antigravity muscle activity decreases due to the combined effects of gravity reduction and neural regulation. With much of the whole body supported by the floor, the gravitational load on antigravity muscles may decrease, potentially reducing the necessity for these muscle’s engagement [12]. Additionally, pressure receptors detect physical contact with the floor and transduction of cutaneous input into the central nervous system. These processes could result in the inhibition of excessive muscle tone and a reduction in postural-related spinal reflex activities, further decreasing muscle activity. These mechanisms collectively suggest a potential explanation for the observed decrease in antigravity muscle activity when supine [14,81,82]. Thus, it is possible that the effectiveness of exercise 1, which increased muscle activity in the abdominal muscles, and exercises 2 and 3, which promoted coordination in the lower extremities for body weight loading, was enhanced by the supine position, which reduced antigravity muscle activity in all exercise programs.

Exercise 1 applied tactile stimulation to multiple locations on the fore surface of the trunk muscles. In general, not all exercise programs that promote localized muscle groups of the trunk, such as the internal obliques and transversus abdominis, touch the lower trunk and feel contractions. Exercise 1 is intended to consciously activate the entire abdominal muscle group, including the local muscles of the trunk. Therefore, exercise 1 may effectively stimulate the contraction of the entire abdominal muscles and improve the ability to control the segmental dynamics of the thorax and pelvis.

No change was observed in the task that required instantaneous maximal muscle strength, whereas improved performance was observed in the task that required continuous shifting of the COG and switching of the direction of motion [23]. Exercises 2 and 3 were mainly designed to promote coordinated muscle activity in the lower extremities during the loading phase in gait. The loading phase dampens the impact at initial contact, which is absorbed by each joint movement of the lower extremities [7]. In addition, the stabilometer task with joined parallel standing imposes substantial balance demands, as maintaining stability for 30 seconds requires continuous sensory integration and fine neuromuscular adjustments involving trunk and lower extremities [10,83]. The results of the stabilometer task indicate that the exercise program enabled participants to sustain more efficient trunk segmental control under this unstable condition, supporting the interpretation that neuromuscular coordination rather than maximal muscle output was the key driver of improvement. In line with this, agility (side-step) and flexibility (long sitting trunk flexion) improved significantly, whereas no significant changes were observed in grip strength, the 50 m run, the long standing jump, or the sit-up. This pattern indicates that the intervention enhanced agility, flexibility, and postural stability through neural control mechanisms, but did not increase maximal strength, explosive power, or muscular endurance. The structure of the exercise program may help explain these results. Trunk stabilization exercises likely improved regulation of the center of mass through enhanced trunk muscle synergy, which is especially critical under reduced BOS [84]. Lower limb coordination exercises may have facilitated smoother trunk–lower extremity integration, enabling efficient balance adjustments. By strengthening sensory feedback and engaging the forefoot, toe exercises may have expanded the functional BOS [85,86]. Although all three exercise types were performed in the supine position, they appear to have induced adaptations in neural control mechanisms that transferred to anti-gravity tasks. Taken together, these findings suggest that the program facilitated more efficient multi-joint and multi-segmental control strategies, resulting in reductions in SA and TLL, as well as improvements in agility and flexibility.In side-step, the intervention significantly decreased TRA/step of the head and thorax, but no significant change was observed in the lower extremities. Thus, it is thought that impact absorption by the lower extremities attenuated the acceleration from the pelvis to the head and enabled stable control of the axial segment. Consequently, it is possible that the exercise program aimed to link the trunk and lower extremities improved the ability to continuously and stably position the head-to-pelvis in spatial orientation in both static and dynamic balance in the antigravitational movement.

Exercise programs such as those employed in this study may be effective as exercise programs for rehabilitation and older adults. Pilates, an exercise method that includes a supine exercise program, improves static and dynamic balance and, clinically, is beneficial for patients with chronic low back pain for 3–9 weeks and < 60 min of Pilates exercise [87]. In this study, the exercise program comprised only three low-impact 10-min programs, which are performed in the supine position and focused on linking trunk stability with lower extremity coordination. The results of this exercise program, which was continued for 2 weeks, showed that it was effective in agility, flexibility, and static standing balance. This result is interesting as it suggests that even the exercise program used in this study, which is performed only in the supine position, can improve body balance and agility performance in antigravitational postures and movements at a relatively safety and short period by linking trunk stability with lower extremity coordination.

However, in this study, no significant differences were observed in grip strength and sit-up tests, which are maximal muscle strength indices, as well as in the long jump test and 50-m run, which are explosive power indices. Regarding the effects of supine-based exercises on muscle strengthening, the finding that 4–24 weeks of Pilates for 45–60 min, mainly mat Pilates in participants aged ≥ 60 years, and the increased muscle strength in the lower extremities [88] suggests that improving muscle power output can benefit from including longer, more prolonged, and higher-impact exercises in the menu.

## Limitations

The exercise program developed in this study emphasized the linkage between the trunk and lower extremities; however, the verification of individual components of the program remains insufficient. Additionally, although key domains of physical fitness were assessed, muscle strength and endurance were not directly measured using detailed physiological instruments, which limits the specificity of the functional interpretations.

For the assessment of dynamic balance, we employed a side-step task that primarily reflects medial–lateral balance ability. This task was selected to emphasize trunk segmental control under repetitive lateral motion involving substantial vertical and horizontal displacement. To evaluate the dynamic responsiveness of high-mass body segments (e.g., pelvis, thorax, and head), we used resultant acceleration as a comprehensive index. However, this approach did not allow for directional decomposition (e.g., anterior-posterior or rotational components). Future studies should incorporate direction-specific analyses, particularly in segments that showed intervention-related changes, to provide a more comprehensive understanding of dynamic postural control.

Moreover, The participants in this study were limited to healthy young adults, which may introduce selection bias and limit the applicability of the findings to populations with balance impairments or comorbidities. Consequently, the results should not be directly extrapolated to older adults or individuals with physical disabilities.

While the experimental design incorporated coreelements of clinical research methodology—such as randomization, control conditions, and structured outcome measures—the primary aim was to investigate the short-term effects of the exercise protocol in a non-clinical context. Accordingly, the findings should be interpreted as foundational data supporting the feasibility of the exercise program, rather than as conclusiveof clinical efficacy. Nevertheless, because the protocol can be performed in a short durationand in a biomechanically stable supine position, it may hold promise for fall prevention or early-phase rehabilitation. Future studies should evaluate its effectiveness in older adults and populations with impaired trunk control.

## Conclusions

This study evaluated the effects of a “supine” exercise program that links head-to-trunk stability with a high mass ratio and lower extremity coordination. This study showed that a short-term complex exercise program effectively improved static and dynamic balance control abilities. Therefore, appropriately implementing an exercise program that links trunk stability and lower extremity coordination is effective in maintaining and improving activities of daily living and QOL. Furthermore, the findings that the number of side-step and kinematic relationships of each axial body segment being practical for evaluating dynamic balance are significant. To validate individual exercise programs and modify the target groups and program duration, further research is warranted.

## Supporting information

S1 File Research plans (Japanese and English), CONSORT checklist, and TREND checklist. (ZIP)

S2 Data Dataset of parameters for Experiment 1 and Experiment 2. (XLSX)
