# Supplementary material for: A supine exercise program linking trunk stability with lower extremity coordination is associated with improved body balance and agility: A study using randomized crossover and pre-post trial designs
Source: PLoS One. 2026 Apr 29;21(4):e0345749. doi: 10.1371/journal.pone.0345749 (PMC13127896; doi:10.1371/journal.pone.0345749)
Supplement: S1 File — (ZIP) [file pone.0345749.s001.zip › S1 Files_revise/Research Plan_Exp 1_Japanese.pdf]

研究計画書

研究課題名

臥位で行う身体チューニングによる立位姿勢及び立位トータルパフォーマンス能力評価

研究の目的及び意義

【研究目的】

以前、我々は、臥位で行う簡単な体操を毎日習慣化し行うことで、体幹の働きが良くなり、立位姿勢の改善のみならず、反復横跳び時に頭部の揺れが抑えられバランス能力がアップすることを見出した。本研究では、立位で行う動作を誘導する臥位で行う体操による、立位トータルパフォーマンス能力を評価する。また、身体の物性変化に着目した計測を行い、パフォーマンス能力変化との関係を調査する。

【研究意義】

近年、腰痛の予防や姿勢・動作の安定性に関わる身体的要因として、体幹機能の重要性が広く認知されてきている。頭部を含めた体幹部の質量比は、身体全体における 60%を占めるとされており、その姿勢制御機能は歩行動作や坐位での日常生活動作における効率性や安定性に大きな影響を与える。立位で行う動作を誘導する臥位で行う体操を習慣化することで、効率性や安定性などのパフォーマンス能力が向上し、日常生活動作に大きな効果をもたらし、心身ともに健康になることが期待される。

研究の方法

全体の工程

・東京農工大学(TUAT)にて臥位で行う体操を行う前に被験者に実験・同意書の説明を行い、同意書のサインと事前アンケートに回答してもらう。本研究では、ランダムクロスオーバー比較試験を行う。

・参加者 2 名ごとの組(ブロック)に介入方法を AB か BA か並べ替えて、2 分の 1 の確率でランダムに割り付けるブロックランダム化によって、被験者を A 群と B 群に振り分ける。

A 群

・東京農工大学にて、「新型コロナウイルス感染症予防対策に関する健康チェックシート」、「日本版 GHQ28 精神健康調査票」に回答してもらい、実験課題および計測項目を実施する(測定 1)。

・その後、臥位で行う体操をレクチャーする。毎日自宅で動画に合わせて臥位で行う体操を行ってもらい、「活動記録と健康状態」を毎日記入してもらう。

・測定 1 から 1 週間後、東京農工大学にて、「新型コロナウイルス感染症予防対策に関する

健康チェックシート」に回答してもらい、体操ができているか個別に確認し、さらに2週目以降の組み合わせ体操を説明する。毎日自宅で動画に合わせて組み合わせ体操を行ってもらい、「活動記録と健康状態」を毎日記入してもらう。

・測定1から2週間後、東京農工大学にて、「新型コロナウイルス感染症予防対策に関する健康チェックシート」、「日本版GHQ28精神健康調査票」に回答してもらい、同様の実験課題および計測項目を東京農工大学で実施する(測定2)。

・臥位で行う体操の効果が持ち越さないように、2週間ウォッシュアウト期間※1を設ける。「活動記録と健康状態」を毎日記入してもらう。

・測定2から2週間後、東京農工大学にて、「新型コロナウイルス感染症予防対策に関する健康チェックシート」、「日本版GHQ28精神健康調査票」に回答してもらい、同様の実験課題および計測項目を東京農工大学で実施する(測定 3)。「活動記録と健康状態」を毎日記入してもらう。

・測定3から2週間後、東京農工大学にて、「新型コロナウイルス感染症予防対策に関する健康チェックシート」、「日本版GHQ28精神健康調査票」に回答してもらい、同様の実験課題および計測項目を東京農工大学で実施する(測定4)。事後アンケートに回答してもらう。

B群

・東京農工大学にて、「新型コロナウイルス感染症予防対策に関する健康チェックシート」、「日本版 GHQ28 精神健康調査票」に回答してもらい、実験課題および計測項目を実施する(測定5)。「活動記録と健康状態」を毎日記入してもらう。

・測定5から2週間後、東京農工大学にて、「新型コロナウイルス感染症予防対策に関する健康チェックシート」、「日本版GHQ28精神健康調査票」に回答してもらい、同様の実験課題および計測項目を東京農工大学で実施する(測定6)。「活動記録と健康状態」を毎日記入してもらう。

・A群と条件を合わせるため、2週間ウォッシュアウト期間を設ける。「活動記録と健康状態」を毎日記入してもらう。

・測定6から2週間後、東京農工大学にて、「新型コロナウイルス感染症予防対策に関する健康チェックシート」、「日本版GHQ28精神健康調査票」に回答してもらい、同様の実験課題および計測項目を東京農工大学で実施する(測定7)。

・その後、臥位で行う体操をレクチャーする。毎日自宅で動画に合わせて臥位で行う体操を行ってもらい、「活動記録と健康状態」を毎日記入してもらう。

・測定7から1週間後、東京農工大学にて、「新型コロナウイルス感染症予防対策に関する健康チェックシート」に回答してもらい、体操ができているか個別に確認し、さらに2週目以降の組み合わせ体操を説明する。毎日自宅で動画に合わせて組み合わせ体操を行ってもらい、「活動記録と健康状態」を毎日記入してもらう。

・測定7から2週間後、東京農工大学にて、「新型コロナウイルス感染症予防対策に関する

健康チェックシート、「日本版 GHQ28 精神健康調査票」に回答してもらい、同様の実験課題および計測項目を東京農工大学で実施する(測定 8)。事後アンケートに回答してもらう。

体操の方法

体操はすべて臥位で行う。また、5 分程度で行えるようになっている。

体操

1)腹筋群の収縮運動：

両膝関節を屈曲し、膝を立てた状態にする。両手で腹部を触り、触った部分の腹筋群を収縮させる。手で押さえる場所を腹部の下方から上方へ、右から左へと変えて、各 1 回ずつ腹部 9 カ所行う。

2)腰椎屈曲および殿筋群の収縮運動

膝を屈曲する。両手は腹部におく。骨盤を後傾させ、その位置で殿部を収縮させる。その後殿部を床からわずかに浮かせる。殿部を 5 秒間収縮しその後 5 秒間リラックスするのを交互に 10 回行う。

3)膝のストレッチ

片脚の足底を床面に接地し、足底を接地し滑らせながら膝を 90°屈曲する。その後、屈曲した脚を足関節背屈、足趾屈曲したまま、踵で床面を滑らせながら股・膝関節を伸展していく。伸展後は、頭尾側方向へ更に踵を伸ばすように下肢全体を伸展することで膝後面筋群のストレッチを 5 秒間行う。下肢は交互に 3 回ずつ行う。

4)足指のストレッチ

足の指でグー・チョキ・パーの動作を行う。図のようにグー・チョキ（親指前）・チョキ（親指後ろ）・パー(図)の順で 20 秒間行う。

被験者には来校時に、体操の画像を見ながら体操の指導を個別に行う。

第 2 回来校時、体操ができているか個別に確認し、さらに 2 週目以降の組み合わせ体操を画像をみながら説明する。

実験課題および計測項目

・個人情報の聴取：年齢、運動経験、メールアドレス、

・身体情報の計測：身長、体重

・実験課題

握力（2 回）

長座体前屈（2 回）

立ち幅跳び（2 回）

上体起こし（30 秒 1 回）
反復横跳び（20 秒 2 回）
50m 走(2 回)
スポーツ庁新体力テスト（20～64 歳対象、2019 年度）に基づき実施する。
以上の計測項目は十分に練習してもらったうえで実施する。
キュートメーター
重心動揺計
スパイロメーター
スパイナルマウス
立位姿勢

計測条件
計測機器
加速度センサ 9 軸加速度センサ（ATR-Promotions の小型無線多機能センサ）
キュートメーター Cutometer®MPA580（Courage+Khazaka 社）、8mm 径プローブ
重心動揺計（竹井機器工業株式会社）
スパイロメーター HI-801（チェスト株式会社）
スパイナルマウス（インデックス社）
デュアルタイプ体組成計 インナースキャンデュアル RD-903（タニタ）
身長計（三和製作所）
50m タイム測定機 FASTRun(ワイワイファクトリー)

動画・画像撮影範囲
各課題にて全身が映る範囲とする。

解析項目
解析方法
・動画解析：
　動画解析ソフトを用い、矢状面※2 および前額面※3 おける頭部・体幹部・骨盤・下肢の平面座標を算出し、空間的位置偏位※4 を検討する。

・画像解析
　画像解析ソフトを用い、体操前後の立位姿勢の比較を行う。t-検定により、有意差があるか解析を行う。

・加速度解析：

加速度センサ貼付部位：頭部・体幹部・骨盤・両足部
頭部・胸郭・骨盤に生じる 3 軸加速度・3 軸角加速度変化[※5]を算出する。

・キュートメーター解析:
　皮膚にプローブをあて陰圧により皮膚を吸引した後、陰圧を解除して皮膚の戻り具合を計測し、粘弾性を測定する。頸部を前屈することで同定できる第 7 頸椎と、腸骨稜を結ぶ線（ヤコビ線）より同定できる第 4 腰椎を基準とする。この 2 つの間を 4 か所測定する。R パラメーター（R0（ハリ）、R5・R7（弾力性）、R6（粘弾性））を算出する。

・重心動揺計解析:
　足圧中心の動揺量から、総軌跡長・面積・速度・ロンベルグ率を算出する。開眼・閉眼条件で、両足と片足立ち 30 秒ずつ行う。

・スパイロメーター解析: 呼吸機能評価
　スパイロメトリーとフローボリューム試験を行う。%肺活量や 1 秒率を算出する。

・スパイナルマウス解析:
　測定機器を背骨に沿って動かし、脊柱の形と可動域を正確に計測する。
矢状面計測：　直立・前屈・後屈の状態で計測する。隣接する椎体間の角度・胸椎後彎角・腰椎前彎角・可動域を算出する。
前額面計測：　直立、左への側屈、右への側屈の状態で計測する。隣接する椎体間の角度・胸椎後彎角・腰椎前彎角・可動域を算出する。

・50m 走の解析
50m 走のタイムを測定する。また、カメラと 10m おきの各通過地点の間に三角コーンを置いて、三角コーンを通過した時のタイムを計測する。これにより、疾走速度・ストライド・ピッチを算出する。

疾走速度
区間距離(10m) を区間タイム(10m を走るのにかかった時間)で除した値。
ピッチとストライドの積で表すことができる。
ピッチ
区間での歩数を区間タイムで除した値。
ストライド
疾走速度をピッチで除した値。

評価
・クロスオーバー試験のため、被験者合計４０名で体操なしとあり（各 n=20）の群間比較で体幹体操の効果の t-検定を行う。
・50m 走のタイムと疾走速度、ピッチ、ストライドの相関解析により、体幹体操により速く走れるようになる説明変数を探る。
・多くの評価項目を設定することで、主成分分析、因子分析、クラスター分析により、体幹体操によりアップする要因（柔軟性・バランス能力・トータル性）を評価する。

予定する研究対象者数
・本研究では　40 例（内訳と詳細は以下）を用いて研究を実施する。
　　＜内訳・試料の詳細＞
　東京農工大学にてホームページ、ポスター等により 40 名を募集

・被験者の選定方針（除外基準）
　十分な判断力のない者は除外する。　日常に臥位で行う体操を行っていない人。やったことがあっても習慣化されていない人は対象とする。

・被験者の種類
20 歳から 25 歳の健常成人男性

研究対象者に生じる負担並びに予測されるリスク及び利益
・研究対象者に生じる負担（心身、時間、経済的負担など）
東京農工大学で行う計測の拘束時間
2 時間程度の拘束時間が生じる。

自宅での臥位で行う体操とアンケート
1 日 10 分程度の拘束時間が生じる。

・研究対象者に生じるリスク（有害事象等）
　反復横跳びや 50m 走の実施時に転倒のリスクが生じる。また、筋肉痛や、筋を痛める恐れもある。
　対面での測定となるため、新型コロナウイルスに罹患する可能性がある。

・リスクを最小化する方法
　実験当日はその日の被験者の体調を確認、必要に応じてバイタルチェックを実施し、被験者が希望した場合はいつでも中止・中断する。

･万が一キャンパス内での計測の際に、具合が悪くなったり怪我をされた場合は、保険管理センターに速やかにご案内をする。状況に応じて速やかに医療機関に連絡して対応する。

新型コロナウイルス感染防止のため、東京農工大学の対応方針に従い、体温測定、アルコール消毒、マスク、換気など適切な行動を心掛ける。また、毎日の健康状態をアンケートの記入と、当日は新型コロナウイルス感染症予防対策に関する健康チェックシートの記入をしてもらう。

験者についても、新型コロナウイルス感染症予防対策に関する健康チェックシートを記入し、感染の可能性が低いことを把握する。

使用機材は、エタノールや次亜塩素酸ナトリウムにより消毒を行う。

予想される利益とリスクを踏まえた総合評価

閉眼条件での立位と歩行時、反復横跳び時に転倒のリスク、筋肉痛や、筋を痛める恐れもあるが、自己のボディバランスを知ることが出来る。また、臥位での体操の効果を実感することができる。

・個々の研究対象者における中止基準

体調不良、有害事象の発生、その他理由を問わず本人からの申し入れ

【研究中止時の対応】

データ破棄の希望を確認し従う、有害事象の発生時は速やかに医療機関に連絡しその指示に従う。

インフォームド・コンセント（IC）を受ける手続き等

被験者には、同意文書および説明文書を用いて、実験の危険性、同意、同意に関する撤回について十分に説明をした上で、文書として同意を得た上で実験を実施する。

研究に用いられる試料・情報の保管及び廃棄の方法

個人情報に関する情報は、東京農工大学 4 号館 238 の施錠可能な棚に厳重に管理し、施錠を徹底する。各データは解析がすべて終了した段階で速やかに破棄する。

個人情報等の取扱い　　□個人情報は取得しない

・　**収集する個人情報**

■氏名　　□住所　　■生年月日

■その他（性別、身長、体重、音声、画像、メールアドレス、運動経験、食習慣）

・　**匿名化方法**

□匿名化しない（説明書・同意書にその旨を記載し提供者の同意を得ること）

■匿名化するが、対応表は作成する
□匿名化し、対応表は作成しない

- **匿名化しない又は対応表を作成する理由**

■提供者の開示又は廃棄の請求に応じる必要がある
■データの解析結果を提供者に知らせる可能性がある
□その他の理由（具体的に記入）

研究対象者等及びその関係者からの相談等への対応

【相談窓口】

渡邊　敏行　東京農工大学　工学研究院　有機材料化学科
〒184-8588　東京都小金井市中町 2-24-16
Tel：042-388-7289
e-mail：toshi@cc.tuat.ac.jp

用語の解説

※1. ウォッシュアウト期間：それまで行っていた体操の効果を排除し、その後の評価を正確に行うために必要な期間のこと。
※2. 矢状面：体を左右に 2 つに分けるように、縦方向に切る断面のこと。
※3. 前額面：身体を腹側と背側の 2 つの部分に分けたときの断面のこと。
※4. 空間的位置偏位：矢状面および全額面における頭部・体幹部・骨盤・下肢がどれだけ動いたか。
※5. 3 軸加速度・3 軸角速度変化：XYZ 軸の 3 方向の加速度。および XYZ 軸に対して角度がどのくらい変化しているか。
