# Supplementary material for: A supine exercise program linking trunk stability with lower extremity coordination is associated with improved body balance and agility: A study using randomized crossover and pre-post trial designs
Source: PLoS One. 2026 Apr 29;21(4):e0345749. doi: 10.1371/journal.pone.0345749 (PMC13127896; doi:10.1371/journal.pone.0345749)
Supplement: S1 File — (ZIP) [file pone.0345749.s001.zip › S1 Files_revise/Research Plan_Exp2_Japanese_revise.pdf]

研究計画書

研究課題名

臥位で行う体操による姿勢改善評価

研究の目的及び意義

【研究目的】

臥位で行う簡単な体操を毎日習慣化し行うことで、日常生活に必要な体幹が身につき、姿勢がよくなることが期待できる。その評価系を構築することが目的である。

【研究意義】

近年、腰痛の予防や姿勢・動作の安定性に関わる身体的要因として、体幹機能の重要性が広く認知されてきている。頭部を含めた体幹部の質量比は、身体全体における60%を占めるとされており、その姿勢制御機能は歩行動作や坐位での日常生活動作における効率性や安定性に大きな影響を与える。臥位で行う体操を習慣化することで、姿勢を改善することができ、日常生活動作に大きな効果をもたらし、心身ともに健康になることが期待されている。しかし、評価方法が確立されていないため、それを構築することが必要とされている。

研究の方法

全体の工程

1. 農工大にて臥位で行う体幹体操を行う前に「参加者事前Webアンケート１」および「測定日健康状態チェック」に回答してもらい、実験課題および計測項目を実施する。
2. その後、体幹体操をレクチャーし、毎日自宅で体幹体操を行ってもらい、記録を毎日記入してもらう。
3. 1週間後、「測定日健康状態チェック」に回答してもらい、1.と同様の計測項目を農工大で実施する。
4. その計測後、体幹体操の理屈や日常で体幹を使うべき場面をレクチャーし、毎日自宅で体幹体操を行ってもらい、どの場面で体幹を使ったかの記録を毎日記入してもらう。
5. 4.の 1 週間後、「測定日健康状態チェック」に回答してもらい、再び同様の計測項目を農工大で実施する。事後アンケートに回答してもらう。

体操の方法

体操はすべて臥位で行う。両手で腹部を触り、触った部分の腹筋を収縮させる。体重を床に預けるような感覚で、腹部の筋を緊張させる。その緊張を維持したままストレッチを行う。

1. 両手で腹部を触り腹筋を収縮させる。

仰向けで寝たまま、膝を屈曲する。この姿勢で両手で腹部を触り、触った部分の腹筋を収

縮させる。腹部を下方から上方へ、右から左へと手で押さえる場所を変えて、腹部全体的に行う。

2.　仰向けで寝て、膝を伸ばす。腰椎を床に付けたまま大腿筋を収縮させる。
腰椎で床を押す感覚で大腿筋を収縮させる。その後、力を抜く。この動作を交互に行う。

3. 仰向けで寝て、片足を床に滑らせるように屈曲し、近位に引き寄せた後、その足の踵を、床面をつま先の方に滑らせながら押し返す。その際、足指を握ったままゆっくりと伸ばす。

4. 仰向けで寝て、足の指でグー・チョキ・パーの動作を行う。

実験課題および計測項目

個人情報の聴取：年齢、性別、利き足、運動経験、メールアドレス、食習慣、音声、画像歩き方

身体情報の計測：身長、体重

姿勢保持課題: 立位姿勢：開眼・閉眼条件（各1分間2回）

片脚立位（バランスマットあり・なし）

両脚立位（開眼条件のみ）

ファンクショナルリーチ（1回）

反復横跳び（20秒1回）＊

握力（2回）＊

長座体前屈（2回）＊

上体起こし（30秒1回）＊

立ち幅跳び（2回）＊

足踏み動作（BPM40,80,120　各1分1回）

*はスポーツ庁新体力テスト（20~64歳対象、2019年度）に基づき実施する。

以上の計測項目は十分に練習してもらったうえで実施する。

計測条件

計測機器

- 9軸加速度センサ5個（ATR-Promotionsの小型無線多機能センサ）（片脚立位、反復横跳び、足踏み動作）
- 動画撮影範囲：各姿勢保持課題・動作課題実施時に矢状面・前額面上にて全身が映る範囲とする
- 加速度センサ貼付部位：頭部・体幹部・骨盤・両足部

解析項目

解析方法

・動画解析：

動画解析ソフトを用い、矢状面※1および前額面※2おける頭部・体幹部・骨盤・下肢の平面座標を算出し、空間的位置偏位※3を検討する。

・画像解析

画像解析ソフトを用い、体操前後の立位姿勢の比較を行う。t−検定により、有意差があるか解析を行う。

・加速度解析：

加速度センサ貼付部位：頭部・体幹部・骨盤・両足部

頭部・胸郭・骨盤に生じる 3 軸加速度・3 軸角加速度変化※4を算出する。

評価 : 課題実施時の加速度センサをつけた部分における垂直性および各体節の加速度・角加速度変化について、開眼条件・閉眼条件の差異を t−検定、各セグメントの加速度・各加速度変化の相関解析などを行い、最終的には重心位置を推定する骨盤部加速度センサのみの場合と、頭部・胸郭部・骨盤部を分けた場合の差異について検討する。

予定する研究対象者数

・ 本研究では 30 例（内訳と詳細は以下）を用いて研究を実施する。

＜内訳・試料の詳細＞

東京農工大学にて 30 名を募集

・ 被験者の選定方針（除外基準）

十分な判断力のない者は除外する。　日常に体幹体操を行っていない人。やったことがあっても習慣化されていない人は対象とする。

・ 被験者の種類

健常成人

研究対象者に生じる負担並びに予測されるリスク及び利益

・ 研究対象者に生じる負担（心身、時間、経済的負担など）

農工大で行う計測の拘束時間：1 回目は 1 時間半程度の拘束時間が生じる。2 回目は 1 時間半程度、3 回目は 1 時間程度の拘束時間が生じる。

自宅で行う体幹体操とアンケート： 1 日 10 分程度の拘束時間が生じる（2 週間）。

・ 研究対象者に生じるリスク（有害事象等）

閉眼条件での立位と歩行時、反復横跳び時に転倒のリスクが生じる。

また、筋肉痛や、筋を痛める恐れもある。

・　リスクを最小化する方法

実験当日はその日の被験者の体調を確認、必要に応じてバイタルチェックを実施し、被験者が希望した場合はいつでも中止・中断する。
万が一キャンパス内での計測の際に、具合が悪くなったり怪我をされた場合は、保険管理センターに速やかにご案内をする。状況に応じて速やかに医療機関に連絡して対応する。

・　予想される利益とリスクを踏まえた総合評価

閉眼条件での立位と歩行時、反復横跳び時に転倒のリスク、筋肉痛や、筋を痛める恐れもあるが、自己のボディバランスを知ることが出来る。また、体幹体操の効果を実感することができる。

・　個々の研究対象者における中止基準

　体調不良、有害事象の発生、その他理由を問わず本人からの申し入れ

　【研究中止時の対応】

データ破棄の希望を確認し従う、有害事象の発生時は速やかに医療機関に連絡しその指示に従う。

インフォームド・コンセント（IC）を受ける手続き等

　被験者には、同意文書および説明文書を用いて、実験の危険性、同意、同意に関する撤回について十分に説明をした上で、文書として同意を得た上で実験を実施する。

研究に用いられる試料・情報の保管及び廃棄の方法

個人情報に関する情報は、東京農工大学 4 号館 238 の施錠可能な棚に厳重に管理し、施錠を徹底する。各データは解析がすべて終了した段階で速やかに破棄する。

個人情報等の取扱い　□個人情報は取得しない

・　収集する個人情報

　■氏名　□住所　■生年月日

　■その他（性別、身長、体重、音声、画像、メールアドレス、運動経験、食習慣）

・　匿名化方法

□匿名化しない（説明書・同意書にその旨を記載し提供者の同意を得ること）

■匿名化するが、対応表は作成する

□匿名化し、対応表は作成しない

・　**匿名化しない又は対応表を作成する理由**

■提供者の開示又は廃棄の請求に応じる必要がある

■データの解析結果を提供者に知らせる可能性がある

□その他の理由（具体的に記入）

研究対象者等及びその関係者からの相談等への対応

【相談窓口】

渡邊　敏行　東京農工大学　工学研究院　有機材料化学科

〒184-8588　東京都小金井市中町 2-24-16

Tel：042-388-7289

e-mail：toshi@cc.tuat.ac.jp

用語の解説

※1.　矢状面：体を左右に 2 つに分けるように、縦方向に切る断面のこと。

※2.　前額面：身体を腹側と背側の 2 つの部分に分けたときの断面のこと。

※3.　空間的位置偏位：矢状面および全額面における頭部・体幹部・骨盤・下肢がどれだけ動いたか。

※4. 3 軸加速度・3 軸角速度変化：XYZ 軸の 3 方向の加速度。および XYZ 軸に対して角度がどのくらい変化しているか。
