# Supplementary material for: A supine exercise program linking trunk stability with lower extremity coordination is associated with improved body balance and agility: A study using randomized crossover and pre-post trial designs
Source: PLoS One. 2026 Apr 29;21(4):e0345749. doi: 10.1371/journal.pone.0345749 (PMC13127896; doi:10.1371/journal.pone.0345749)
Supplement: S1 File — (ZIP) [file pone.0345749.s001.zip › S1 Files_revise/Research Plan_Exp 1_English.pdf]

Research Plan

Title of Research

Evaluation of Standing Posture and Physical Performance After Supine Exercises

Purpose and Significance of the Study

Purpose of the study

Previously, we found that daily practice of simple gymnastics exercises performed in the supine position improves not only the trunk function and standing posture, but also the balance ability by suppressing head sway during jumping. In this study, we will evaluate the standing total performance ability by exercises performed in the supine position, which induce movements performed in the standing position. In addition, measurements focusing on changes in physical properties of the body will be conducted to investigate the relationship with changes in performance ability.

Significance of the Study

In recent years, the importance of the trunk function has been widely recognized as a physical factor related to the prevention of back pain and stability of posture and movement. The mass ratio of the torso, including the head, is estimated to account for 60% of the total body mass, and its postural control function has a significant impact on efficiency and stability in walking and seated daily activities. It is expected that habitual practice of exercises performed in the supine position, which induces movements similar to those performed in the standing position, will improve performance abilities such as efficiency and stability, and have a significant impact on daily living activities, resulting in better physical and mental health.

Research Methodology

Overall process

- This study is designed as a randomized crossover trial. Prior to the intervention involving supine exercises at Tokyo University of Agriculture and Technology (TUAT), participants will receive an explanation of the study and complete the informed consent process, including signing the consent form and responding to a pre-intervention questionnaire.
- Participant allocation will be conducted using block randomization. Pairs of participants (blocks of two) will be randomly assigned to one of two intervention sequences (AB or BA) with an equal allocation ratio (1:1).

Group A

- Participants will be asked to answer the “Health Check Sheet for Prevention of New Coronavirus Infections” and the “Japanese GHQ28 Mental Health Questionnaire” at TUAT and will perform the experimental tasks and measurement items (Measurement 1).
- Afterwards, a lecture will be given on exercises to be performed in the supine position. The participants will be asked to perform the exercises at home daily in the supine position according to a video, and to fill in the “Activity Record and Health Status” daily.
- One week after Measurement 1, the participants will be asked to fill out the “Health Check Sheet for Prevention of New Coronavirus Infections” at TUAT, and individually checked to see if they are doing the exercises. We ask them to perform the combination exercises at home daily according to videos and fill in the “Activity Record and Health Status” daily.
- Two weeks after Measurement 1, the same experimental tasks and measurement items will be conducted at TUAT (Measurement 2) by having the participants complete the “Health Check Sheet on Preventive Measures against New Coronavirus Infections” and the “Japanese GHQ 28 Mental Health Questionnaire” at TUAT.
- Provide a 2-week washout period so that the effects of the exercises performed in the supine position do not carry over. Have the residents fill out the “Activity Record and Health Status” daily.
- Two weeks after Measurement 2, participants will be asked to complete the “Health Check Sheet for Prevention of New Coronavirus Infections” and the “Japanese GHQ28 Mental Health Questionnaire” at TUAT, and the same experimental tasks and measurement items will be conducted at TUAT (Measurement 3). Two weeks after Measurement 3, the participants will be asked to fill in the “Activity Record and Health Status” daily.
- Two weeks after Measurement 3, participants will be asked to complete the “Health Check Sheet for Prevention of New Coronavirus Infections” and the “Japanese GHQ28 Mental Health Questionnaire” at TUAT, and the same experimental tasks and measurement items will be conducted at TUAT (Measurement 4). They will be asked to respond to a post-event questionnaire.

Group B

- The participants will be asked to complete the “Health Check Sheet on Preventive Measures against New Coronavirus Infections” and the “Japanese GHQ 28 Mental Health Questionnaire” at TUAT to perform the experimental tasks and measurement items (Measurement 5). The participants will be asked to fill in the “Activity Record and Health Status” daily.

- Two weeks after Measurement 5, participants will be asked to complete the “Health Check Sheet for Prevention of New Coronavirus Infections” and the “Japanese GHQ28 Mental Health Questionnaire” at TUAT and complete the same experimental tasks and measurement items at the university (Measurement 6). The participants will be asked to fill in the “Activity Record and Health Status” daily.
- A 2-week washout period will be conducted in order to match the conditions of A. (A two-week washout period will be conducted to match the conditions of Group A. Participants will be asked to complete the “Activity Record and Health Status” daily.
- Two weeks after Measurement 6, participants will be asked to complete the “Health Check Sheet for Prevention of New Coronavirus Infections” and the “Japanese GHQ28 Mental Health Questionnaire” at NITH, and the same experimental tasks and measurement items will be conducted at NITH (Measurement 7).
- They will then be lectured on exercises to be performed in the supine position. The participants will be asked to perform the exercises at home daily in the supine position according to a video, and to fill out the “Activity Record and Health Status” daily.
- One week after the measure 7, the participants will be asked to answer the “Health Check Sheet on Prevention Measures for New Coronavirus Infections” at TUAT, and individually check whether they are doing the exercises, and further explain the combination exercises for the second week onward. We will ask them to perform the combination exercises at home daily according to videos and fill in the “Activity Record and Health Status” daily.
- Two weeks after Measurement 7, the participants will be asked to complete the “Health Check Sheet on Preventive Measures for New Coronavirus Infections” and the “Japanese GHQ28 Mental Health Questionnaire” at TUAT, and the same experimental tasks and measurement items will be conducted at TUAT (Measurement 8). They will be asked to respond to a post-event questionnaire.

Method of Exercises

All exercises are performed in the supine position. They can also be performed in about 5 minutes.

Exercises:

1. Both knee joints are flexed, and the knees are placed in an upright position. Touch the abdomen with both hands and contract the abdominal muscles in the touched area. Change the location of the abdomen to be touched with the hands from downward to upward and from right to left and perform each exercise once in each of 9 locations on the abdomen.

2. Flex the knees. Place both hands on the abdomen. Tilt the pelvis backward and contract the gluteus maximus in that position. Then, raise the hallux slightly off the floor. Contract the gluteus maximus for 5 seconds and then relax for 5 seconds, alternately, 10 times.
3. With the sole of one leg on the floor, bend the knee 90° while sliding the sole of the foot on the floor. Then, with the flexed leg dorsiflexed and the toes flexed, extend the hip and knee joints while sliding on the floor with the heel. After extension, the posterior knee muscle group is stretched for 5 seconds by extending the entire lower limb as the heel is further extended in a cephalocaudal direction. The lower extremities are alternately stretched 3 times each.
4. Perform the Rock-Paper-Scissors motion with the toes. As shown in the figure, perform Rock, Scissors (thumb forward), Scissors (thumb backward), and Paper in this order for 20 seconds.

When the participants visit the school, they are individually instructed on the exercises while watching the images of the exercises.
At the second visit to the school, the participants will be individually checked to see if they are doing the exercises, and the combination exercises for the second and subsequent weeks will be explained to them while viewing the images.

Experimental tasks and measurement items
Personal information: age, exercise experience, and e-mail address,
Measurement of physical information: height, weight
Experimental tasks:
Grip strength (2 times)
Sitting trunk flexion (2 times)
Standing long jump (2 times)
Sit-ups (once for 30 seconds)
Side-step (2 times for 20 seconds)
50-meter run (2 times)
The above Items will be conducted based on the Sports Agency's new physical fitness test (for 20-64 year olds, FY2019).
The above measurement items will be conducted after sufficient practice.
Cutometer
Stabilometer
Spirometer
Spinal Mouse

Standing posture

Measurement conditions

Measurement equipment

Accelerometer 9-axis accelerometer (ATR-Promotions' compact wireless multifunction sensor)

Cutometer®MPA580 (Courage+Khazaka), 8mm diameter probe

Stabilometer (Takei Kiki Kogyo Co., Ltd.)

Spirometer HI-801 (Chest Corporation)

Spinal Mouse (Index Corporation)

Dual Type Body Composition Meter InnerScan Dual RD-903 (Tanita Corporation)

Height meter (Sanwa Seisakusho)

50-m time measuring machine FASTRun (YY Factory)

Recording Scope for Video and Still Images:

The entire body should be captured in each task.

Analysis Items

Analysis Method

Video analysis:

Using video analysis software, the plane coordinates of the head, torso, pelvis, and lower limbs in the sagittal plane[*2] and forehead plane[*3] are calculated to examine spatial positional deviation[*4].

Image analysis

Using image analysis software, the standing posture before and after the exercise will be compared. t-test will be used to analyze whether there is a significant difference.

Acceleration analysis:

Acceleration sensor attachment sites: head, torso, pelvis, and both legs.

Calculate the triaxial acceleration and triaxial angular acceleration changes[*5] that occur in the head, thorax, and pelvis.

Cutemeter analysis:

After applying a probe to the skin and suctioning the skin by negative pressure, the negative pressure is released, and the return of the skin is measured to measure viscoelasticity. The seventh cervical vertebra, which can be identified by bending the neck forward, and the fourth lumbar vertebra, which can be identified by the line connecting the iliac crest (Jacobi line), are used as standards. Four points between these two will be

measured; R parameters (R0 (firmness), R5 and R7 (elasticity), and R6 (viscoelasticity)) will be calculated.

Center-of-gravity sway meter analysis:.

Calculate total locus length, area, velocity, and Romberg rate from the amount of foot pressure center sway. Both feet and one leg standing for 30 seconds each under open and closed eye conditions.

Spirometry analysis: evaluation of respiratory function

Perform spirometry and flow volume test. Calculate % lung capacity and 1 second rate.

Spinal Mouse Analysis:.

Move the measuring instrument along the spine to accurately measure the shape and range of motion of the spinal column.

Sagittal plane measurement: Measurements are taken in upright, forward bending, and backward bending positions. The angle between adjacent vertebrae, thoracic kyphosis angle, lumbar kyphosis angle, and range of motion are calculated.

frontal plane measurement: Measurements are taken in upright, left lateral flexion, and right lateral flexion. Calculate the angle, thoracic kyphosis angle, lumbar kyphosis angle, and range of motion between adjacent vertebrae.

Analysis of 50m run

The time of the 50m run is measured. A triangular cone is placed between the camera and each passing point every 10 m, and the time when the participant passes through the triangular cone is measured. The running speed, stride, and pitch are then calculated.

sprinting speed: The value obtained by dividing the distance (10m) by the section time (time taken to run 10m). It can be expressed as the product of pitch and stride.

Pitch: Value obtained by dividing the number of steps taken in a segment by the segment time.

Stride: The value obtained by dividing the speed of the sprint by the pitch.

Evaluation

A t-test will be conducted on the effect of the physical exercises on the runners with and without gymnastics (n=20 each) for a total of 40 participants for a crossover study.

The correlation analysis of 50m running time, sprinting speed, pitch, and stride will be used to determine the explanatory variables for the effect of the bodyweight exercises on running speed.

By setting up many evaluation items, we will evaluate the factors (flexibility, balance ability, and total performance) that are improved by physical exercises through principal component analysis, factor analysis, and cluster analysis.

Number of subjects to be studied

Forty participants will be used in this study. 40 participants will be recruited at TUAT via posters, etc.

Selection policy for subjects (exclusion criteria)

Participants who lack sufficient judgment will be excluded. Those who do not perform daily exercises in the supine position. Those who have done them before but have not made it a habit will be included.

Types of subjects

Healthy adult males between the ages of 20 and 25 years old.

Burden on research subjects and anticipated risks and benefits

Burden on research subjects (physical, mental, time, financial, etc.)
Time required for measurements at TUAT will be approximately 2 hours.
Exercises and questionnaires to be performed in the supine position at home.
About 10 minutes of restraining time per day will be required.

Risks to the study subjects (adverse events, etc.)

There is a risk of falling during the repetitive horizontal jump and 50-meter run. There is also a risk of muscle pain and sore muscles.
There is a possibility of contracting a new type of coronavirus due to the face-to-face measurement.

Methods to minimize risk

On the day of the experiment, the participant's physical condition for the day will be checked, vitals will be checked if necessary, and the experiment will be stopped or suspended at any time if the participant requests.
-In the unlikely event that a subject becomes ill or injured while taking measurements on campus, the insurance administration center will be notified immediately. Contact a medical facility as soon as possible to deal with the situation.
To prevent transmission of the new coronavirus, we will follow the response policy of the College of Agriculture and Technology, and take appropriate actions such as taking body temperature, disinfecting with alcohol, wearing a mask, and ventilating the room. In addition, the participants will be asked to fill out a daily health questionnaire and a health

check sheet regarding measures to prevent new-type coronavirus infection on the day of the study.
The participants will also be asked to fill out a health check sheet regarding measures to prevent new coronavirus infection, to ascertain that there is a low probability of infection. Equipment used should be disinfected with ethanol or sodium hypochlorite.

Overall evaluation based on anticipated benefits and risks.
Although there is a risk of falling, muscle soreness, or even muscle damage during standing and walking under closed-eye conditions and during side-step, the participants will be able to learn about their own body balance. Also, the participants will be able to feel the effects of the body exercises.

・Discontinuation criteria in individual study subjects
Discontinuation due to physical condition, occurrence of adverse events, or any other reason upon request from the individual subject.

[Response to discontinuation of research]
In the event of an adverse event, the medical institution will be promptly notified and instructions will be followed.

Methods of Storage and Disposal of Samples and Information Used in Research
Information related to personal information will be kept strictly in a lockable cabinet in TUAT Building No. 4, room238, and will be securely locked. Each data will be destroyed as soon as all analyses are completed.

Procedures for obtaining informed consent (IC), etc.
Experiments will be conducted after participants have been fully informed of the risks of the experiment, consent, and withdrawal of consent by means of consent and explanation documents, and after their written consent has been obtained.

Handling of Personal Information, etc.
□We do not collect personal information.
• Personal information to be collected
■Name □Address ■Date of birth
■Other (gender, height, weight, voice, image, e-mail address, exercise experience, eating habits)

• Anonymization method

□Not anonymized (This should be stated in the explanation and consent form and the consent of the donor should be obtained.)

■Anonymize, but create a correspondence table.

□Anonymize, but do not create a correspondence table.

• Reasons for not anonymizing or creating a correspondence table

■It is necessary to respond to the provider's request for disclosure or disposal of the data.

■There is a possibility that the results of the data analysis will be communicated to the donor.

□Other reasons (please specify)

Response to Consultations, etc., from Research Subjects, etc., and Parties Involved

[Consultation Desk]

Toshiyuki Watanabe
Department of Organic Materials Chemistry, Faculty of Engineering, Tokyo University of Agriculture and Technology
2-24-16 Nakamachi, Koganei-shi, Tokyo 184-8588, Japan
Tel: 042-388-7289
e-mail: toshi@cc.tuat.ac.jp

Explanation of Terms

*1. washout period: a period necessary to eliminate the effects of the previously performed exercises and to accurately conduct subsequent evaluations.

*2. Sagittal plane: A section cut longitudinally so that the body is divided into two halves, left and right.

*3. Anterior forehead plane: a cross-sectional section of the body cut so that it is divided into two parts, ventral and dorsal.

*4. Spatial position deviation: The degree of movement of the head, torso, pelvis, and lower limbs in the sagittal and full-face planes.

*5. Triaxial acceleration and triaxial angular velocity change: Acceleration in the three directions of the XYZ axes. and how much the angles have changed in relation to the XYZ axes.
