# Supplementary material for: A supine exercise program linking trunk stability with lower extremity coordination is associated with improved body balance and agility: A study using randomized crossover and pre-post trial designs
Source: PLoS One. 2026 Apr 29;21(4):e0345749. doi: 10.1371/journal.pone.0345749 (PMC13127896; doi:10.1371/journal.pone.0345749)
Supplement: S1 File — (ZIP) [file pone.0345749.s001.zip › S1 Files_revise/Research Plan_Exp 2_English_revise.pdf]

Research Plan

Title of Research

Improvement Effects of Standing Posture After Supine Exercises

Purpose and Significance of the Study

Purpose of the study

It is expected that the daily practice of simple exercises performed in the supine position will help the patients acquire the core strength necessary for daily living and improve their posture. The purpose of this study is to establish an evaluation system.

Significance of the Study

In recent years, the importance of the trunk function has been widely recognized as a physical factor related to the prevention of back pain and stability of posture and movement. The mass ratio of the torso, including the head, is estimated to account for 60% of the total body mass, and its postural control function has a significant impact on efficiency and stability in walking and seated daily activities. It is expected that the habitual practice of exercises performed in the supine position, which induces movements performed in the standing position, will improve performance abilities such as efficiency and stability, and have a significant effect on daily living activities, resulting in better physical and mental health. However, since no evaluation method has been established, it is necessary to build one.

Research Methodology

Overall process

1. Before the participants perform the supine exercises at the Tokyo University of Agriculture and Technology (TUAT), we will ask them to answer the "Participants' Preliminary Web Questionnaire 1" and "Health Status Check on the Day of Measurement" and perform experimental tasks and measurement items.
2. After that, the participants will be given a lecture on exercises and asked to perform the exercises at home daily and fill out a daily record.
3. One week later, the participants will be asked to answer the "health status check on the day of measurement," and the same measurement items as in 1. will be conducted at TUAT.
4. After the measurement, we will give them a lecture on the logic behind the exercises and situations in which they should use their trunk in their daily lives and ask them to perform the exercises at home daily and fill in a daily record of which situations they have used

their trunk.

5. One week after 4., we will ask the participants to answer the “Measurement Day Health Status Check” and conduct the same measurement items again at TUAT. They will be asked to respond to a post-measurement questionnaire.

Methods of Exercises

All exercises are performed in the supine position. Touch the abdomen with both hands and contract the abdominal muscles in the touched area. Tense the abdominal muscles, as if you were placing your weight on the floor. Stretch while maintaining that tension.

1. Touch the abdomen with both hands and contract the abdominal muscles.
   While lying on your back, flex your knees. In this position, touch the abdomen with both hands and contract the abdominal muscles in the touched area. Change the position of the abdomen from downward to upward and from right to left and perform the exercise over the entire abdomen.
2. While lying on your back, flex your knees. Contract the thigh muscles while keeping the lumbar spine attached to the floor. Contract the thigh muscles as if pressing the floor with the lumbar spine. Then relax. Perform this action alternately.
3. Lying on your back, flex one leg as if sliding it on the floor, pull it proximally, and then push the heel of that leg back while sliding it across the floor toward your toes. As you do so, hold the toes and stretch them slowly.
4. Lying on your back, perform the Rock-Paper-Scissors motion with your toes.

Experimental tasks and measurement items

Interviewing personal information: age, gender, dominant foot, exercise experience, e-mail b address, eating habits, voice, picture walking style

Measurement of physical information: height, weight

Posture maintenance task

Standing posture: Eye opening and eye closing conditions (2 times for 1 minute each)
One-leg standing posture (with and without balance mat)
Both legs standing (eye-open condition only)

Functional reach (1 time)

Side-step (1 time for 20 seconds) *

Grip strength (2 times) *

Sitting trunk flexion (2 times) *

Sit-ups (1 time for 30 seconds) *

Standing long jump (2 times) *
Foot stomping motion (BPM 40, 80, 120, 1 time for 1 minute each)
* Items will be conducted based on the Sports Agency's new physical fitness test (for 20~64-year-olds, FY2019).
The above measurement items will be conducted after sufficient practice.

Measurement conditions

Measurement device:

- Five 9-axis acceleration sensors (ATR-Promotions small wireless multifunctional sensors) (one-leg standing, Side-step, and foot stomping movements)
- Range of video recording: The whole body in the sagittal and forehead planes during each posture holding and movement task.
- Location of accelerometers: head, thorax, pelvis, and both feet

Analysis Items

Video analysis:

・Using video analysis software, the plane coordinates of the head, torso, pelvis, and lower limbs in the sagittal plane[※1] and forehead plane[※2] were calculated, and spatial positional deviation[※3] was examined.

・Acceleration analysis:

Calculate triaxial acceleration and triaxial angular acceleration changes[※4] that occur in the head, thorax, and pelvis.

Evaluation ： Conduct t-tests for differences between the open-eye and closed-eye conditions for verticality and acceleration and angular acceleration changes in each body segment at the part with the accelerometer when performing the task, as well as correlation analysis of acceleration and each acceleration change in each segment.

Finally, we will examine the differences between the case of the pelvic accelerometer alone, which estimates the center of gravity position, and the case of the head, thorax, and pelvic segments separately.

Number of subjects to be studied

Thirty participants will be used in this study. 30 participants will be recruited at TUAT.

Selection policy for subjects (exclusion criteria)

Participants who do not have sufficient judgment will be excluded. Those who do not perform body exercises daily. Those who have done them before but have not made it a habit will be included.

Type of subjects
Healthy adults

Burden to Research Subjects and Anticipated Risks and Benefits
Burden on research subjects (physical, mental, time, financial, etc.)
Time required for measurements to be taken at the University of Agriculture and Technology
The first measurement will take about 1.5 hours; the second measurement will take about 1.5 hours; and the third measurement will take about 1 hour.
Body exercises to be done at home and questionnaire. A time constraint of 10 minutes per day will be imposed. (2 weeks)

Risks to study subjects (adverse events, etc.)
There is a risk of falling during standing and walking in the closed-eye condition and during side-step.
There is also a risk of muscular pain and muscle damage.

Methods to minimize risk
On the day of the experiment, the participant's physical condition for the day will be checked, vitals will be checked if necessary, and the experiment will be stopped or suspended at any time if the participant requests.
-In the unlikely event that a participant becomes ill or injured while taking measurements on campus, the insurance administration center will be notified immediately. Depending on the situation, contact a medical institution as soon as possible to deal with the situation.

Overall evaluation based on anticipated benefits and risks.
Although there is a risk of falling, muscle soreness, or even muscle damage during standing and walking under closed-eye conditions and during side-step, the participants will be able to learn about their own body balance. Also, the participants will be able to feel the effects of the body exercises.

・Discontinuation criteria for individual research subjects
Discontinuation due to illness, adverse events, or any other reason upon request from the

individual subject.

[Response to discontinuation of research]
In the event of an adverse event, the medical institution will be promptly notified, and instructions will be followed.

Methods of Storage and Disposal of Samples and Information Used in Research
Information related to personal information will be kept strictly in a lockable cabinet in TUAT Building No. 4, room238, and will be securely locked. Each data will be destroyed as soon as all analyses are completed.

. Procedures for obtaining informed consent (IC), etc.
Experiments will be conducted after participants have been fully informed of the risks of the experiment, consent, and withdrawal of consent by means of consent and explanation documents, and after their written consent has been obtained.

Handling of Personal Information, etc.
□We do not collect personal information.
・Personal information to be collected
■Name □Address ■Date of birth
■Other (gender, height, weight, voice, image, e-mail address, exercise experience, eating habits)
・Anonymization method
□Not anonymized (This should be stated in the explanation and consent form and the consent of the donor should be obtained.)
■Anonymize but create a correspondence table.
□Anonymize, but do not create a correspondence table.

・Reasons for not anonymizing or creating a correspondence table
■It is necessary to respond to the provider's request for disclosure or disposal of the data.
■There is a possibility that the results of the data analysis will be communicated to the donor.
□Other reasons (please specify)

Response to Consultations, etc., from Research Subjects, etc., and Parties Involved

[Consultation Desk]
Toshiyuki Watanabe
Department of Organic Materials Chemistry, Faculty of Engineering, Tokyo University of Agriculture and Technology
2-24-16 Nakamachi, Koganei-shi, Tokyo 184-8588, Japan
Tel: 042-388-7289
e-mail: toshi@cc.tuat.ac.jp

Explanation of Terms

※1. Sagittal plane: A section cut longitudinally so that the body is divided into two halves, left and right.

※2. Anterior forehead plane: a cross-sectional section of the body cut so that it is divided into two parts, ventral and dorsal.

※3. Spatial position deviation: The degree of movement of the head, torso, pelvis, and lower limbs in the sagittal and full-face planes.

※4. Triaxial acceleration and triaxial angular velocity change: Acceleration in the three directions of the XYZ axes. and how much the angles have changed in relation to the XYZ axes.
